# Linking statistical shape models and simulated function in the healthy adult human heart

**DOI:** 10.1371/journal.pcbi.1008851

**Published:** 2021-04-15

**Authors:** Cristobal Rodero, Marina Strocchi, Maciej Marciniak, Stefano Longobardi, John Whitaker, Mark D. O’Neill, Karli Gillette, Christoph Augustin, Gernot Plank, Edward J. Vigmond, Pablo Lamata, Steven A. Niederer

**Affiliations:** 1 Cardiac Electromechanics Research Group, Biomedical Engineering Department, King´s College London, London, United Kingdom; 2 Cardiac Modelling and Imaging Biomarkers, Biomedical Engineering Department, King´s College London, London, United Kingdom; 3 Cardiovascular Imaging Department, King’s College London, London, United Kingdom; 4 Department of Cardiology, St Thomas’ Hospital, London, United Kingdom; 5 Institute of Biophysics, Medical University of Graz, Graz, Austria; 6 Institute of Electrophysiology and Heart Modeling, Foundation Bordeaux University, Bordeaux, France; 7 Bordeaux Institute of Mathematics, University of Bordeaux, Bordeaux, France; University of Michigan, UNITED STATES

## Abstract

Cardiac anatomy plays a crucial role in determining cardiac function. However, there is a poor understanding of how specific and localised anatomical changes affect different cardiac functional outputs. In this work, we test the hypothesis that in a statistical shape model (SSM), the modes that are most relevant for describing anatomy are also most important for determining the output of cardiac electromechanics simulations. We made patient-specific four-chamber heart meshes (*n* = 20) from cardiac CT images in asymptomatic subjects and created a SSM from 19 cases. Nine modes captured 90% of the anatomical variation in the SSM. Functional simulation outputs correlated best with modes 2, 3 and 9 on average (*R* = 0.49 ± 0.17, 0.37 ± 0.23 and 0.34 ± 0.17 respectively). We performed a global sensitivity analysis to identify the different modes responsible for different simulated electrical and mechanical measures of cardiac function. Modes 2 and 9 were the most important for determining simulated left ventricular mechanics and pressure-derived phenotypes. Mode 2 explained 28.56 ± 16.48% and 25.5 ± 20.85, and mode 9 explained 12.1 ± 8.74% and 13.54 ± 16.91% of the variances of mechanics and pressure-derived phenotypes, respectively. Electrophysiological biomarkers were explained by the interaction of 3 ± 1 modes. In the healthy adult human heart, shape modes that explain large portions of anatomical variance do not explain equivalent levels of electromechanical functional variation. As a result, in cardiac models, representing patient anatomy using a limited number of modes of anatomical variation can cause a loss in accuracy of simulated electromechanical function.

## Author summary

The heart adapts to physiological and pathological changes in loading. This can cause the heart to change size and shape. These changes can in turn have significant impact on cardiac function. However, it is not clear if large changes in function are caused by large changes in shape or can smaller changes also be important. Biophysical computational models of the heart provide a quantitative framework for mapping changes in anatomy to whole heart function. We created a publicly available healthy four-chamber heart virtual cohort from clinical images. Each patient’s heart anatomy in the virtual cohort was described by the contribution of different components of heart shape. The shape components are ranked by the amount of shape variance that they explain. Simulations of cardiac electrical activation and mechanical pump function in hearts with shapes described by different combinations of shape components were performed. This allowed us to show that some shape components that explain a large amount of electrical and mechanical function variance only explain a small amount of anatomical variance. This highlights the need to have high fidelity anatomical models in cardiac simulations and demonstrates that subtle changes in cardiac anatomy can have a large impact on cardiac function.

## 1 Introduction

Cardiac anatomy plays a crucial role in determining the function of the heart [1–3]. However, mapping changes in anatomy to functional cardiac phenotypes remains challenging. Patient-specific computational models provide a quantitative framework for linking anatomy to cardiac function [4–8]. In patient-specific cardiac models, the anatomy is often derived from Computed Tomography (CT), echocardiography or Magnetic Resonance Imaging (MRI) [9]. Differences in imaging modality will impact the accuracy of the anatomical model of a specific patient’s heart [10], adding an extra layer of uncertainty [11]. These different imaging modalities have known biases [12, 13], yet we do not know how specific changes in the observed cardiac anatomy will impact subsequent simulations of cardiac function.

Cardiac anatomy can be efficiently encoded through Statistical Shape Models (SSM) [14]. These SSM can be created by first registering multiple anatomies onto a single representative average mesh and then finding a compact representation of the registration fields. Principal Component Analysis (PCA) is the most common dimensionality reduction technique used to find the orthogonal directions of deformation (or modes). As a result, each cardiac anatomy can be reconstructed as a linear combination of the anatomical modes that deform the average mesh. The coefficients of the linear combinations of each mesh (sometimes referred to as scores or weights) provide a succinct description of shape and can be used as biomarkers for stratification [15–18], quantifying shape uncertainty [11] or to generate synthetic models [19]. The modes can be ordered according to the amount of variance they explain: the first modes explain most of the shape variance, and latter modes describe more subtle shape differences. Identifying which modes have the greatest impact on cardiac function can identify the anatomical variations that are most important for determining specific cardiac function.

The first modes are most likely to be consistent between imaging modalities [12]. If simulated cardiac function was more dependent on these modes, the model’s functional predictions would be more likely to be independent of image modality. Simulations of cardiac function may also depend on latter modes that describe specific local shape variations, making simulations of cardiac function dependent on the anatomical detail present in each imaging modality used to construct the anatomical model. In this case, focused imaging of specific anatomical features may lead to images that can be used to make more precise models or predictors of specific cardiac function.

In this study, we make four main contributions. First, we describe the creation and quality assurance tests of a virtual cohort of 20 hearts from asymptomatic patients, made publicly available online. Second, we study the relationship between cardiac electromechanical (EM) function and variation in shape modes, identifying the most relevant shape modes for EM simulations. Third, we perform a global sensitivity analysis (GSA) using Gaussian processes emulators (GPEs) to quantify the variance explained in EM function by each one of the anatomical modes. Fourth, we test the effect that several parameters of the EM simulations have on the simulations’ output through a local sensitivity analysis (LSA).

## 2 Methods

Firstly, we describe the image acquisition protocol and the pipeline we built to generate four-chamber meshes from CT images. We then present the pipeline to build the SSM and the quality checks we performed on all the generated meshes. Secondly, we describe the functional simulations we ran and the statistical tests to relate generated anatomy to simulated function. Lastly, we present the sensitivity analyses, both global and local, to further understand the interaction between anatomy and simulated function.

### 2.1 Ethics statement

The imaging data were collected as part of a prospective study approved by the Health Research Authority (18/LO/1803). The study conformed with the Declaration of Helsinki (reference ID 15/LO/1803) and all participants provided written, informed consent.

### 2.2 Creating the anatomical model cohort from CT images

Healthy control static cardiac CT images were obtained from 20 asymptomatic patients at St. Thomas’ Hospital in London, United Kingdom. These patients went to the emergency room with acute chest pains. Since no cardiac conditions were detected in follow-up, these patients were taken as representative of “healthy” (or asymptomatic) hearts. CT images were acquired, 71.35% ± 3.57% through the R-R wave interval, during the diastolic phase. In- and out-plane resolution were 0.34 ± 0.03 mm and 0.49 ± 0.02 mm, respectively.

**Fig 1:**
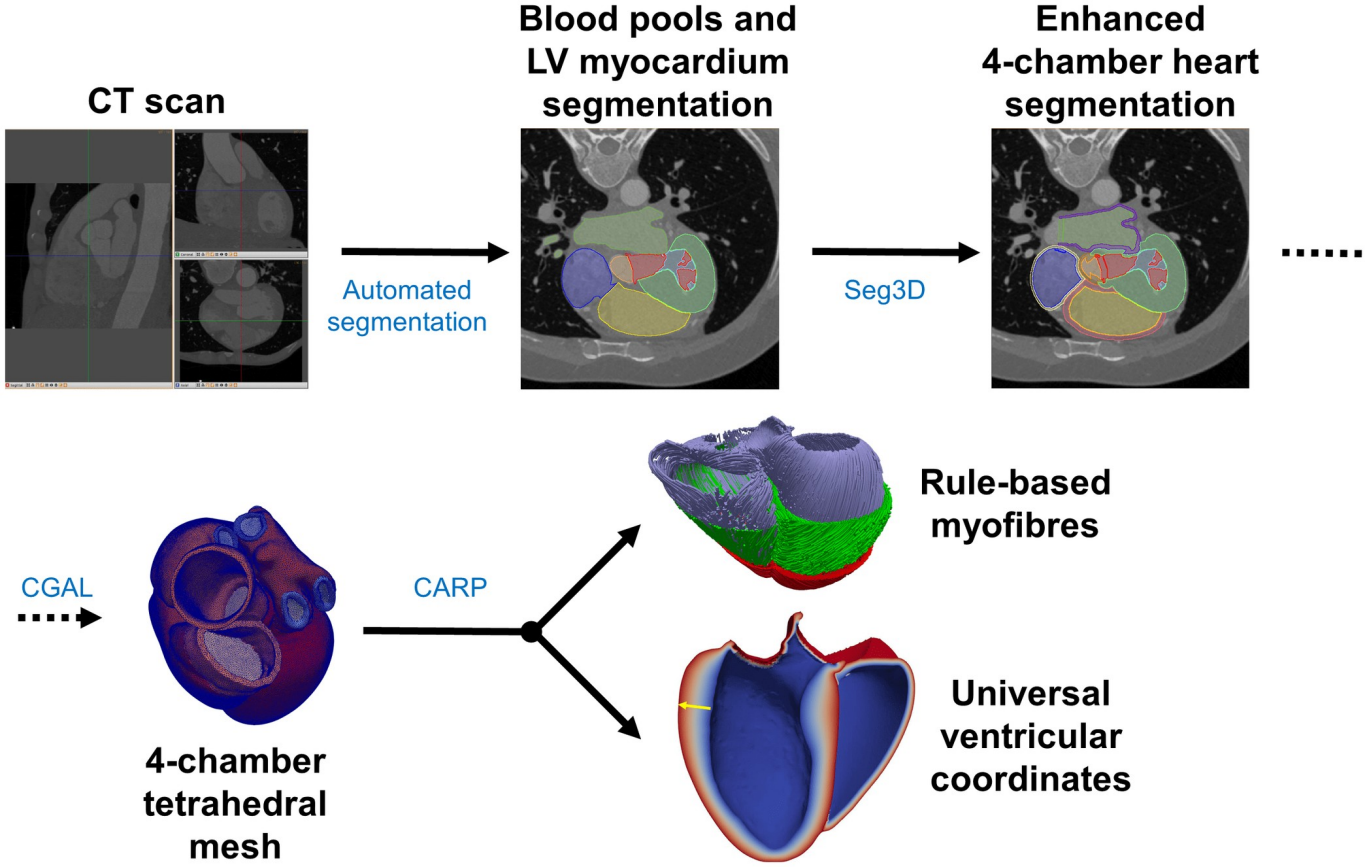
Schematic pipeline of the CT cohort creation, from CT scan to the final end-diastolic mesh including fibres UVC. More details on each one of the steps can be found in S1 and S2 Text.

The methods for mesh construction have been described previously [20]. Briefly, we segmented four-chamber hearts from CT images using an automatic segmentation step [21] with post-processing using Seg3D [22]. The final segmentation consisted of 31 different labels for the blood pools, myocardium and the outflow tracts of the main vessels as well as the papillary muscles. The cardiac valves were modelled as surfaces between the blood pools of the chambers. Similar surfaces were also added to the vessel locations to close the endocardial surfaces. We built unstructured tetrahedral meshes of all labels but the blood pools and papillary muscles using the Computational Geometry Algorithm Library (CGAL) [23] with average edge length of 1 mm. One of the main differences of our meshes with respect to other whole-heart meshes, as in [24], is the addition of auxiliary anatomical components needed to add mechanics boundary conditions. These include the outflow tracts of several cardiac vessels, as well as veins inlet/outlet surfaces. We assigned rule-based fibres to the ventricles [25] as well as a system of Universal Ventricular Coordinates [26] (UVC) using the Cardiac Arrhythmia Research Package (CARP) [27, 28]. A summary of this pipeline can be found in Fig 1 and more details are provided in S1 and S2 Text.

We then calculated the volume using the 3D Stokes theorem approach, described previously in [29]. We quantified mesh element quality using the scaled Jacobian (SJ) metric [30].

### 2.3 Creating the synthetic cohort from a SSM

We built a SSM from the anatomical meshes using the method described in [31]. In short, we rigidly aligned the meshes and extracted the surfaces, representing them as deRham currents [32] to avoid the need of point-to-point correspondence. Intuitively, the current of a surface is the flux of any 3-D vector field across that surface [33]. The registration between meshes and computation of the average shape (also called atlas or template) was done using a Large Deformation Diffeomorphic Metric Mapping method [32, 34]. This method finds simultaneously the average mesh and the deformations functions to warp this average mesh into each one of the meshes. The deformation functions depend on a set of uniformly distributed control points in which the shapes are embedded, and on the deformation vectors attached to these points. It is in this spatial field of deformation vectors (one per each control point) where the PCA is applied. In contrast to deforming the mesh in a pointwise manner, this method deforms the whole space containing the meshes, through the control points. The SSM was built from 19 cases: case 20 was discarded since it could not be aligned due to significantly different morphology of the left atrium and the left pulmonary veins (PVs). More information on the details can be found in S3 Text.

We created two extra cohorts by modifying the weight of the modes explaining 90% of the variance in shape. We created these meshes with either ±2 or ±3 standard deviations (SD) of each mode added to the average mesh (extreme2 and extreme3 cohorts respectively). We also created two additional meshes with ± 1 SD for mode 2 (extreme1 cohort), since the simulations in the more extreme meshes failed to complete.

We refer to the average mesh, extreme1, extreme2 and extreme3 cohorts as the synthetic cohort/meshes. As before, we added fibres and UVC to each mesh. Detailed information about the process can be found in S3 Text.

### 2.4 Cardiac function simulations

We ran EM simulations using CARP [27, 28] as described previously [20, 35]. Briefly, we used the reaction-eikonal model for electrophysiology (EP) [36] and we simulated large deformation mechanics based on [28].

We simulated the effect of a Purkinje system by adding a fast endocardial conduction (FEC) layer [37], covering up to the 70% of the apico-basal distance. The rest of the myocardium we modelled it as a transversely isotropic material.

To simulate the active stress (more details on S4 Text), we used the phenomenological activation-based Tanh Stress model [38, 39].

As initial conditions for the mechanics we used prescribed pressures in the ventricles, in the aorta and in the pulmonary artery. We used omni-directional spring boundary conditions on the superior pulmonary veins in the left atrium and on the superior vena cava in the right atrium. In a similar way as done in [35], we included pericardium boundary conditions. Briefly, we constrained the normal displacement of the epicardium of the ventricles to allow downward displacement of the atrioventricular plane. We scaled the stiffness of the pericardium such that the apex has the maximum penalty and the base (defined through the UVC) has free motion.

We modelled the ventricles as hyperelastic materials following the Guccione’s material law [40] while all the other structures were modelled as neo-Hookean materials [41]. Only the ventricles contracted, while all the other structures were set as passive.

For the circulatory system, we used two three-element Windkessel models, one for the systemic circulation and one for the pulmonary circulation to represent ventricular afterload [42]. In these models, conceptually similar to electrical circuits, valves are represented as resistances to the flow. The arteries are represented with an element of resistance, to take into account the friction, working in parallel with a capacitor representing arterial compliance.

We ran all simulations using the same material properties, initial conditions and boundary conditions to isolate the impact of anatomical variance on simulation predictions. More details on the boundary conditions, preload and afterload as well as parameters values of the different models used can be found in S4 Text. We analysed simulation outputs of the left ventricle (LV) and right ventricle (RV) function in terms of changes in volume (volume-based), changes in pressure (pressure-based), changes in phase duration (time/duration-based) and changes in activation times (EP-based). The full list of EM measurements and outputs with their corresponding abbreviations are in Table 1. All values are derived from the meshes, as opposed to the images. We computed the EM measurements for both ventricles individually except for QRS, AT1090 and LV total activation time. First-Phase Ejection Fraction is defined as the percentage of change in LV volume from end-diastole to the time of peak aortic valve flow. This novel biomarker has been hypothetised to be an early indicator of aortic stenosis and HFpEF [43].

**Table 1:** Measurements taken from the meshes and the EM simulations, with the acronyms used in the text and plots. The first group corresponds to volume-based metrics, the second to pressure-based, third to time/duration-based and the bottom group to EP-based metrics.

Abbrevation	Meaning
ESV	End systolic volume
SV	Stroke volume
EF	Ejection fraction
V1	Volume at time of peak flow
EF1	First-Phase Ejection Fraction
ESP	End-systolic pressure
dPdtmax	Maximum increase of pressure
dPdtmin	Maximum decrease of pressure
PeakP	Peak pressure
tpeak	Time to peak pressure
ET	Ejection time
ICT	Isovolumic contraction time
IRT	Isovolumic relaxation time
tsys	Duration of systole
QRS	QRS duration
AT1090	Time taken to activate from 10% to 90% of the mesh
AT	Activation time of the left ventricle

### 2.5 Study of the relationship between anatomy and simulated function

We performed a GPE-based GSA as in [44]. Briefly, we trained one GPE per phenotype defined in Table 1, with the exception of phenotypes whose range of variability was below a threshold, determined below, across the cohort. The input dataset consisted of 51 vectors of 18 components. Each vector represented the corresponding mesh, while each component represented each modes’ weights. The same input dataset was used for each phenotype GPE. Only the meshes whose simulations completed were included in the dataset. We performed the training by leave-one-out cross-validation of the CT cases. At each split, the training dataset always included all the synthetic cases. The GPE accuracy was measured using the mean squared error (MSE) computed on the left-out point. We estimated Sobol’ sensitivity first-order indices and total effects [45] through the Saltelli method [46] using the SALib Python library [47]. The best scoring GPE (corresponding to the one which achieved the smallest MSE on the validation set) was evaluated at Sobol’ quasi-random sequences made of (2*D* + 2) × *N* points (in our case, *D* = 18 and *N* = 1000).

We performed the statistical analyses in R [48] and generated the plots using the package corrplot [49].

### 2.6 Impact of non-SSM parameters

To asses the influence of the main model parameters and on the simulation results, we carried out a local sensitivity analysis on the average mesh. We modified the values of parameters involved in the mesh construction as well as related to the different submodels used in the EM simulations.

We quantified the role of the atria by using only a biventricular model, tunning the stiffness of the spring-like boundary conditions in the pericardium to achieve the same EF as with the presence of the atria (57.3%).

We modified fibre orientation from the default values of *α*_endo_ = 80° to *α*_epi_ = −60° at the endocardium to epicardium, respectively, to either *α*_endo_ = 75° to *α*_epi_ = −55° or *α*_endo_ = 85° to *α*_epi_ = −65°. We will refer to these configurations as default, narrow or wide. We run simulations using the narrow configuration in both ventricles, only in the LV and only in the RV and using the wide configuration in both ventricles, only in the LV and only in the RV. In the ventricle not modified we used the default fibres.

We tested if the FEC layer was important by introducing a fractal Purkinje network [50] on the apical 70% of the endocardium. For this experiment we used the same CVs as with the FEC model and left all the other parameters with their default value as in the original code [50]. We tested if the fibre CV or FEC layer CV impacted results by modifying them by ±10%. We tested the importance of the aortic and pulmonary afterload by altering the corresponding resistance by ±10%. We also modified the initial and boundary conditions by altering LV and RV end-diastolic pressure (initial conditions) and the Robin/spring boundary condition stiffness by ±10%. We tested the impact of the mechanics model by altering the peak isometric tension, the ventricle passive mechanics scaling parameter *a* (see S4 Text) and the atrial and vein stiffness parameter *c*_atria_ by ±10%.

As a measure of sensitivity for the continuous variables we used the slope of the segment containing the two new pair of parameter-output values normalised. Let (*x*_0_, *y*_0_), (*x*_−_, *y*_−_), (*x*_+_, *y*_+_) be the original input-output, and the modified values respectively where *x*_−_ = *x*_0_ − 10%*x*_0_ and *x*_+_ = *x*_0_ + 10%*x*_0_. Then we define the sensitivity coefficient *SC* as

$$\begin{array}{c} SC=\frac{x_{0}}{y_{0}}\cdot \frac{y_{+}-y_{-}}{x_{+}-x_{-}}. \end{array}$$ (1)

Assuming linearity, if this value is close to ±1 means that a change of 10% in the input value leads to a change in ±10% of the output value. The closer to 0, the less sensitive it is.

## 3 Results

### 3.1 CT cohort

**Fig 2:**
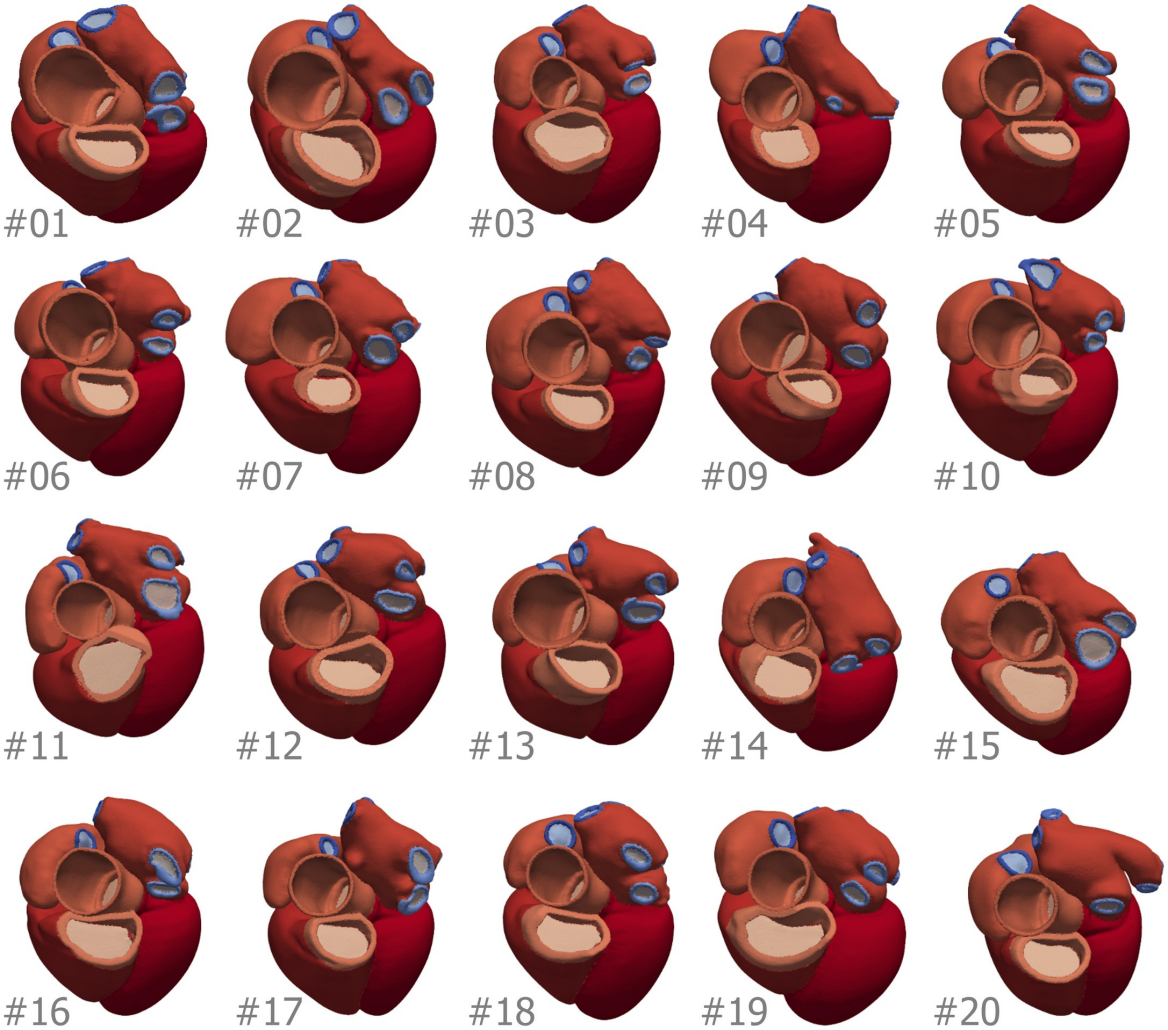
Meshes of the 20 cases of the CT cohort, with the number of the case in the bottom left corner of each image. Colour indicate mesh labels described in S1 Text and referenced in the tables in S5 Text. For the SSM construction we discarded mesh #20.

The CT cohort consisted of 14 males and 6 females, mean age 51 ± 8 years old, ranging from 37 to 67 years and a weight of 85 ± 19 kg ranging from 60 to 146 kg. See Table 2 for the specific demographics of each subject. All the meshes of the CT cohort are shown in Fig 2.

**Table 2:** Demographics of the patients undergoing CT scans. NA stands for not available.

Patient #	Sex	Age	Weight (kg)
01	M	57	66
02	M	49	101
03	M	37	95
04	M	39	92
05	F	59	60
06	M	54	80
07	F	50	95
08	M	47	NA
09	M	67	73
10	M	47	79
11	M	56	146
12	M	57	72
13	M	49	90
14	F	41	90
15	F	48	85
16	M	60	86
17	M	55	74
18	F	50	76
19	F	47	NA
20	M	43	78
**Total**	30% F	51 ± 8	85 ± 19

**Fig 3:**
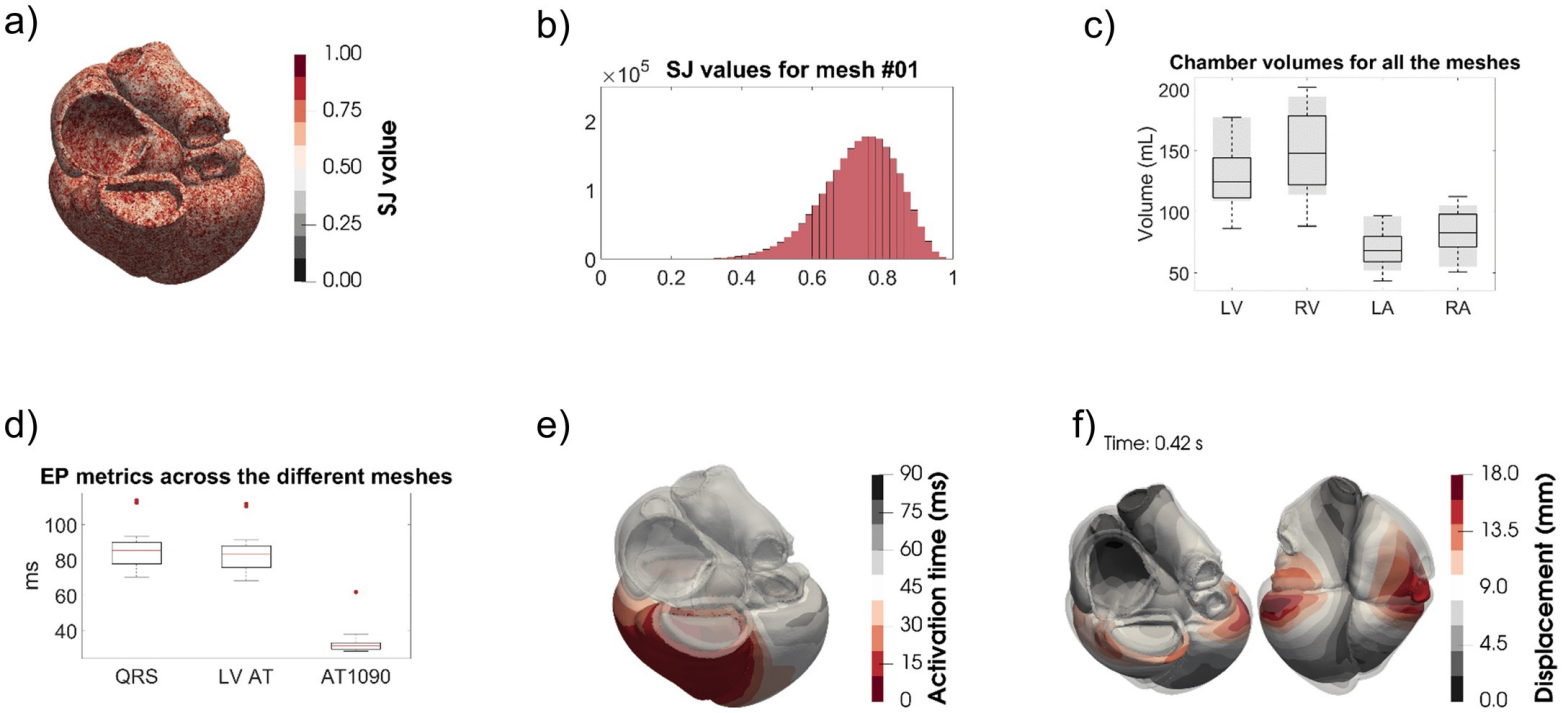
The different quality checks done on the meshes. a) Example of the SJ values projected in a mesh (#01). b) The histogram corresponding to the SJ values of a). c) The blood pool volumes compared with those in the UK Biobank (shaded grey). d) The activation times as described in the text. e) An example of the activation times (each isochrone corresponding to 10 ms). f) The displacement of the mechanics simulation (in translucid the end-diastolic configuration) for mesh #01.

An example of the SJ values projected onto a mesh and as a histogram are shown in Fig 3A and 3B respectively (both corresponding to mesh #01). In S5 Text we provide the values of the SJ, edge lengths and number of elements, nodes and edges for the CT and extreme3 cohorts.

We compared the anatomical volumes of the CT cohort with the values from the UK Biobank in a population cohort of over 5000 individuals [51]. We show the results in Fig 3C where the grey region is the mean ± SD of the results in the UK Biobank. If we stratify by age and sex, the volumes of our CT cohort fall in the lower part of the distributions provided in [51]. For the LV mass we have taken the volume of the LV mesh as the sum of the volume of its elements and multiplied it by a myocardial density of 1.05 g/mL [52], obtaining mass values of 139.68 ± 35.34 g, consistent with the results reported in literature (158 ± 56.8 g) [53].

### 3.2 Statistical shape analysis

**Fig 4:**
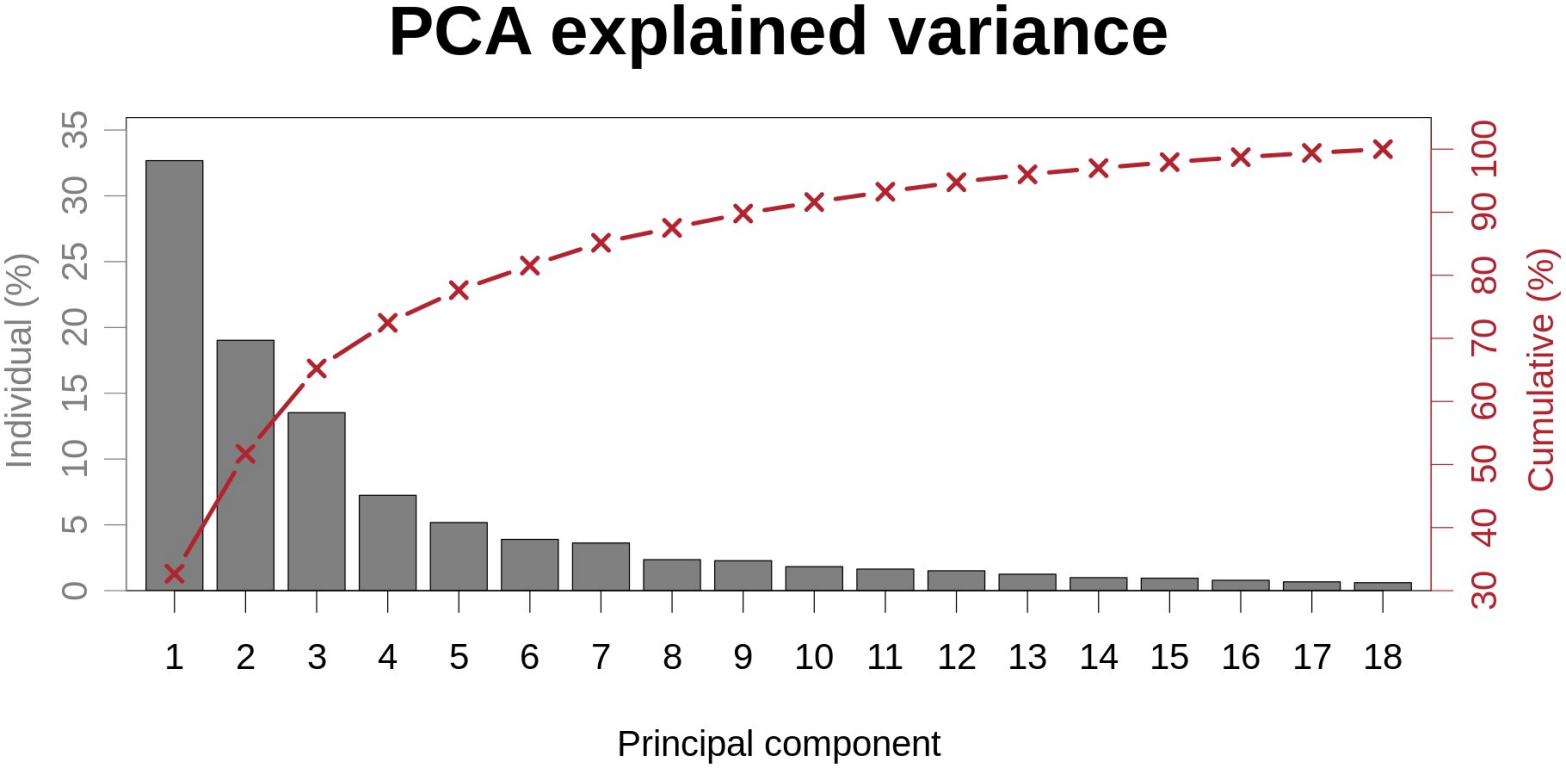
Cumulative and individual explained variance ratio of each PCA mode in the CT cohort.

The percentage of variance in shape explained by each PCA component is shown in Fig 4. The variance in the CT cohort of each mode is normalised over the total variance of all the modes weights. The first 2 components accounted for the majority of the variance (51.70%). The first 9 modes explained 89.99% of the variance.

**Fig 5:**
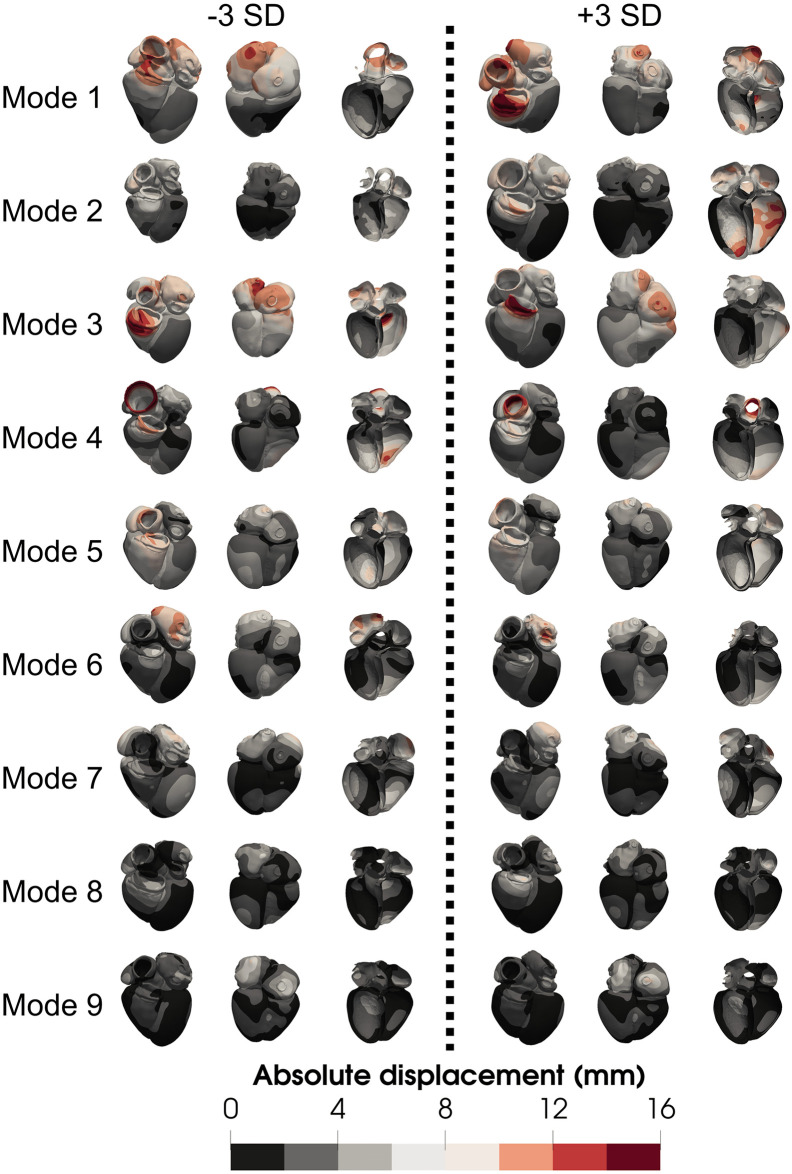
Visual representation of the first 9 modes of the PCA, scaled. Meshes correspond to the extreme3 cohort colour-coded with their distance with respect to the average mesh.

To provide a visualisation of the modes, we show a distance map for modes 1 to 9 in Fig 5. We used the same colour scale for all the meshes to highlight the difference in magnitude between the first and the latter modes, where small deformations could be confounded with noise.

The first mode, explaining most of the anatomical variance, does not alter the size of the heart, or of any of the chambers, but the relative position and orientation of each chamber with respect to each other. It also affects the orientation of the heart (notice the pulmonary artery in the anterior view, or the apex in the inferior view). Similar effects are reflected in modes 3 and 7 as well. Mode 2 is the main mode accounting for difference in size. We notice that the difference in chamber volume is not followed by a similar change in LV wall thickness, creating a wall thinning when the chamber is more dilated. Low values in mode 5 achieve a more spherical heart (globally).

Several modes affect the shape of the LV. A more conic shape of the chamber is achieved decreasing mode 4, creating a less spherical apex. Mode 9 accounts for the thickening of the base, creating a bulge right below the aortic valve. With regards to the RV, increments on mode 3 create an increased basal diameter, pushing the lateral wall outwards. A similar effect is also observed with alterations in mode 7.

Changes in the atria include mode 3, creating a more spherical RA, and pushing it closer to the RV. Mode 6 modifies the size of the LA, creating a smaller chamber with higher values of this mode. Changes in anatomy of the aorta and PA are driven by changes in mode 4 and mode 8 respectively. Mode 7 also modifies its shape, making it less spherical, but also reduces the size of the RA.

### 3.3 Simulated function

EP simulations took 32.98 ± 16.87 seconds on a desktop machine using 20 cores. We ran mechanics simulations on ARCHER (http://www.archer.ac.uk/), the UK national high-performance computing service located at the University of Edinburgh. Before the cardiac cycle, an unloading step was needed to achieve an estimate of the stress-free reference configuration [54] since the meshes were in ventricular diastole. This step took 17.46 ± 2.84 minutes on 168 cores. The cardiac cycle simulation took 8.84 ± 3.18 hours on 432 cores.

Boxplots for the QRS duration, LV AT and AT1090 together with a visual example of the simulated activation pattern on mesh #01 are shown in Fig 3D. Simulated QRS duration ranges from 70 − 114 ms, consistent with reported values of 40 − 120 ms [55]. An example of the activation times of one of the cases is shown in Fig 3E.

Details on the EM simulations can be found in S4 Text. For the CT cohort, the mean ESV for the LV is 55.28 ± 10.64 mL, the stroke volume is 70.19 ± 13.57 mL and the ejection fraction (EF) is 55.91% ± 2.38%. These results are comparable with the ones reported from the UK Biobank [51] of mean ESV of 58 ± 17 mL, stroke volume of 85 ± 20 mL and EF of 60% ± 6%. In Fig 3F the simulated end-systolic configuration for mesh #01 is shown in comparison to the end-diastolic state (translucent grey mesh).

Large deformation mechanics simulations require the solution of a nonlinear system of differential equations. No convergence is guaranteed in such complex geometries and this is a known and persistent problem in cardiac mechanics simulations [9]. Overall 88% of simulations completed. In the CT cohort 90% of simulations completed, with meshes 9 and 10 failing to complete. In the extreme3 cohort 77.78% completed, 4 meshes simulations did not complete, corresponding to mode 2 − 3 SD, mode 3 + 3 SD, mode 6 − 3 SD and mode 9+ 3 SD. In the extreme2 cohort 94.44% completed, mode 2 − 2 SD simulation did not complete. Since in both extreme cohorts a modification of mode 2 simulations did not complete, we created two extra meshes modifying mode 2 ± 1 SD, in which cases both simulations completed. In all the aforementioned cases where the simulations did not complete, RV was close to reaching peak pressure. A summary of the convergence of simulations can be found in Table 3. More details on mesh quality assessment and simulations can be found in S4 Text.

**Table 3:** Summary of the finished simulations for each cohort and in total. SD stands for standard deviation.

Cohort		Total simulations finished			Failed cases
CT		18/20 (90%)		52/59 (88.14%)	#9, #10
Synthetic	Average	1/1 (100%)	34/39 (87.18%)		-
	Extreme3	14/18 (77.78%)			Mode 2 − 3 SD, mode 3 + 3 SD, mode 6 − 3 SD, mode 9 + 3 SD
	Extreme2	17/18 (94.44%)			Mode 2 − 2 SD
	Extreme1	2/2 (100%)			-

### 3.4 Identifying simulation phenotypes with limited anatomical dependence

**Fig 6:**
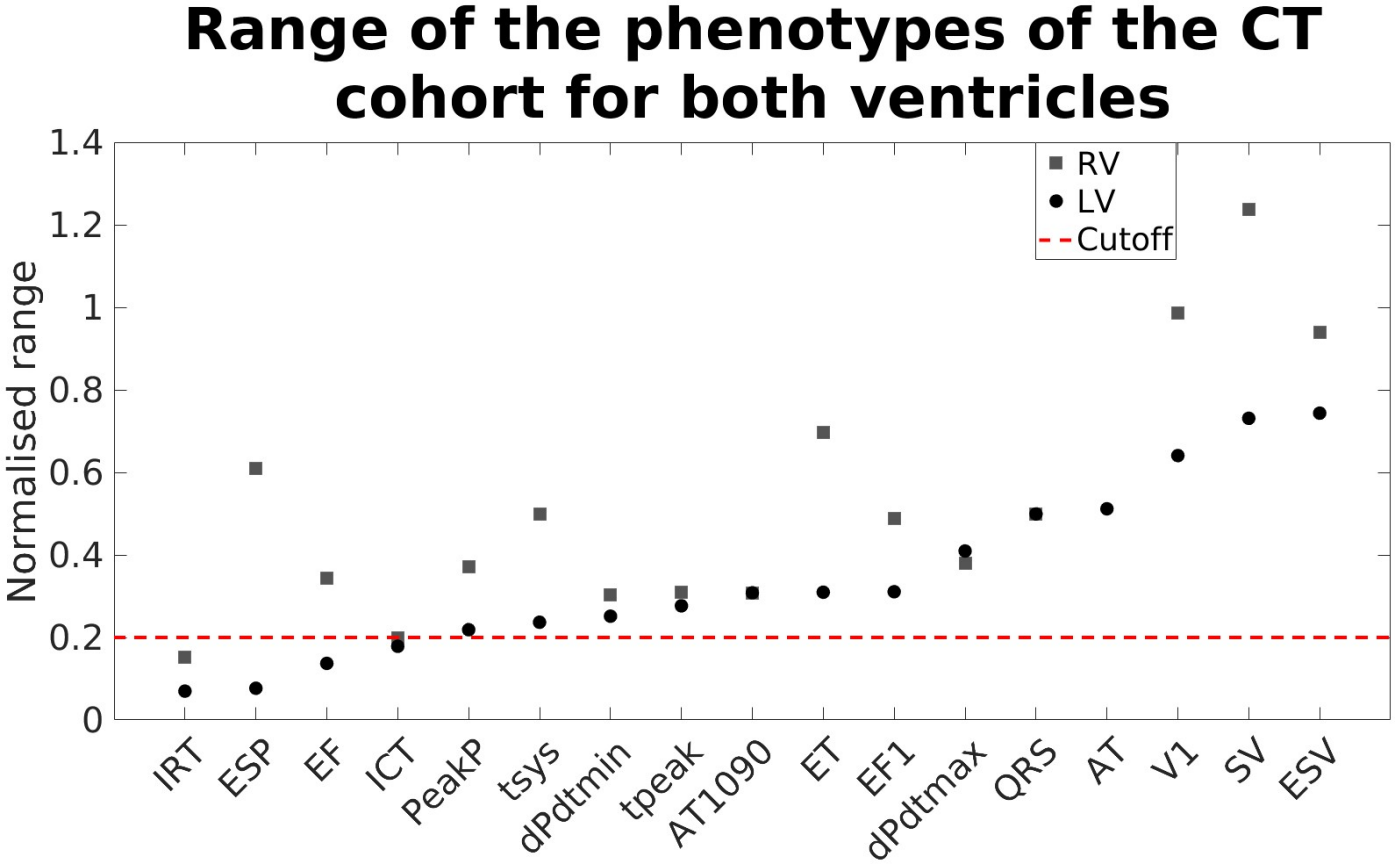
Range covered by each one of the simulated phenotypes of the CT cohort for both ventricles, normalised with the average value of each one of them. The discontinuous red line marks the threshold chosen to discard the simulated phenotypes. In these cases, the range of functional change was considered too small for further study.

To assess which functional phenotypes are less dependent on shape, we measured the range of the phenotypes of the CT cohort normalised by the mean value of each phenotype. This gives the maximum percentage change of each phenotype over the anatomies in the CT cohort. In Fig 6 we show the different values for both ventricles. To allow us to focus on simulation outputs that were affected by shape we discarded phenotypes with a variation below 0.2. This included the LV and RV isovolumic relaxation time and isovolumic contraction time and LV end-systolic pressure and EF phenotypes.

### 3.5 Identifying shape modes that correlate with simulated phenotypes

**Fig 7:**
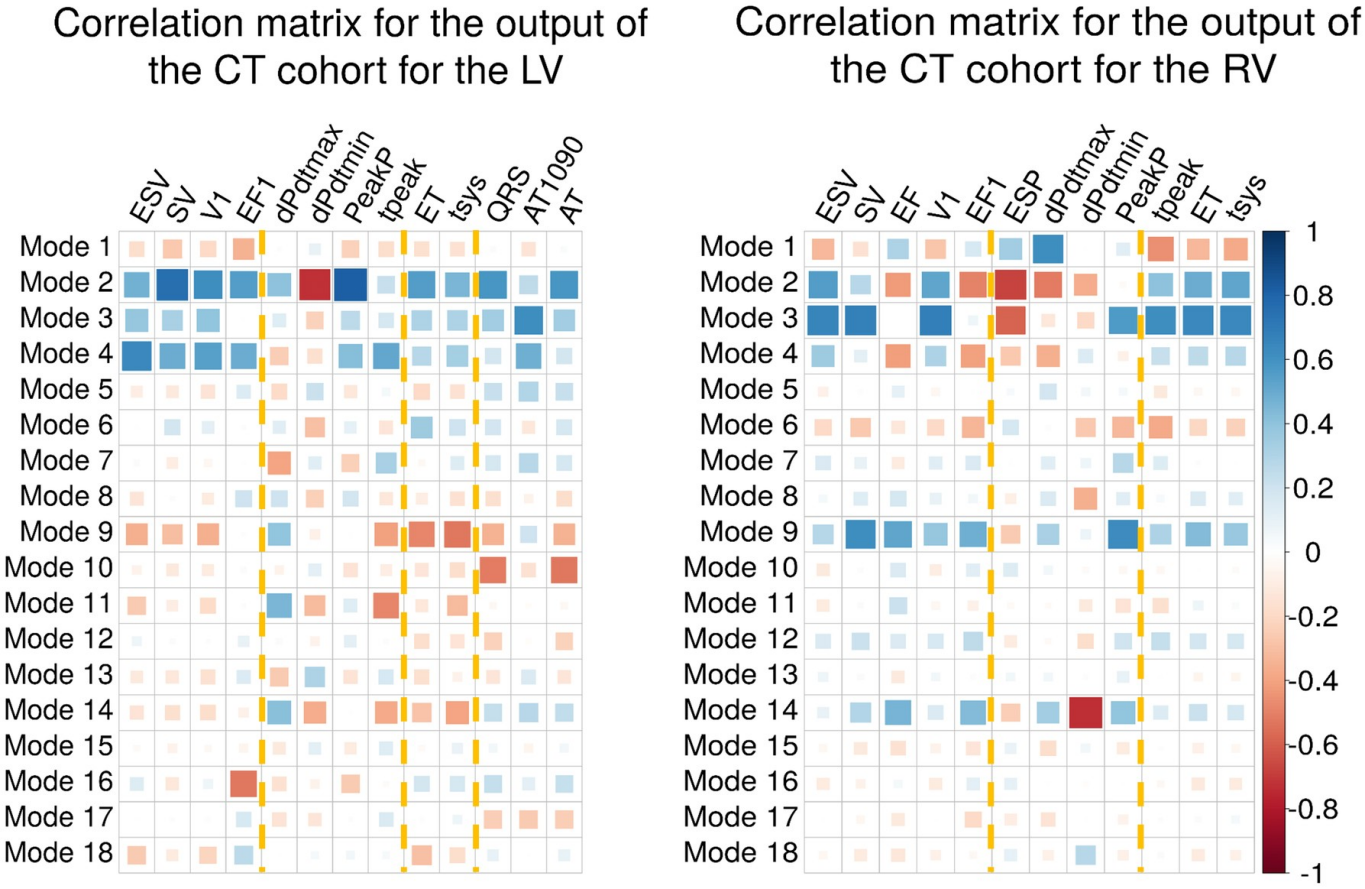
Correlation matrices for the CT cohort. Measurements for the LV on the left, and for the RV on the right. Colour indicates the sign of the correlation and the size of the squares how high is the correlation. Yellow dotted lines indicate each group of phenotypes: volume-based, pressure-based, time/duration-based and EP-based.

To study which modes are the most relevant for each one of the simulated EM outputs, we computed the correlation between each mode and each phenotype in the CT cohort (Fig 7). The most relevant modes are mode 2 and mode 9. Mode 2, that explained 19.02% of variance in the shape field, has the highest average absolute correlation of *R* = 0.49 ± 0.17. In the case of mode 9, although it explains less than 3% of shape variation, had an average absolute correlation of *R* = 0.34 ± 0.17, the highest after modes 2 and 3. The average of the absolute correlation of all the other modes, ranged from 0.08 ± 0.04 (mode 15) to 0.38 ± 0.22 (mode 3).

### 3.6 Identifying modes that impact groups of phenotypes

**Fig 8:**
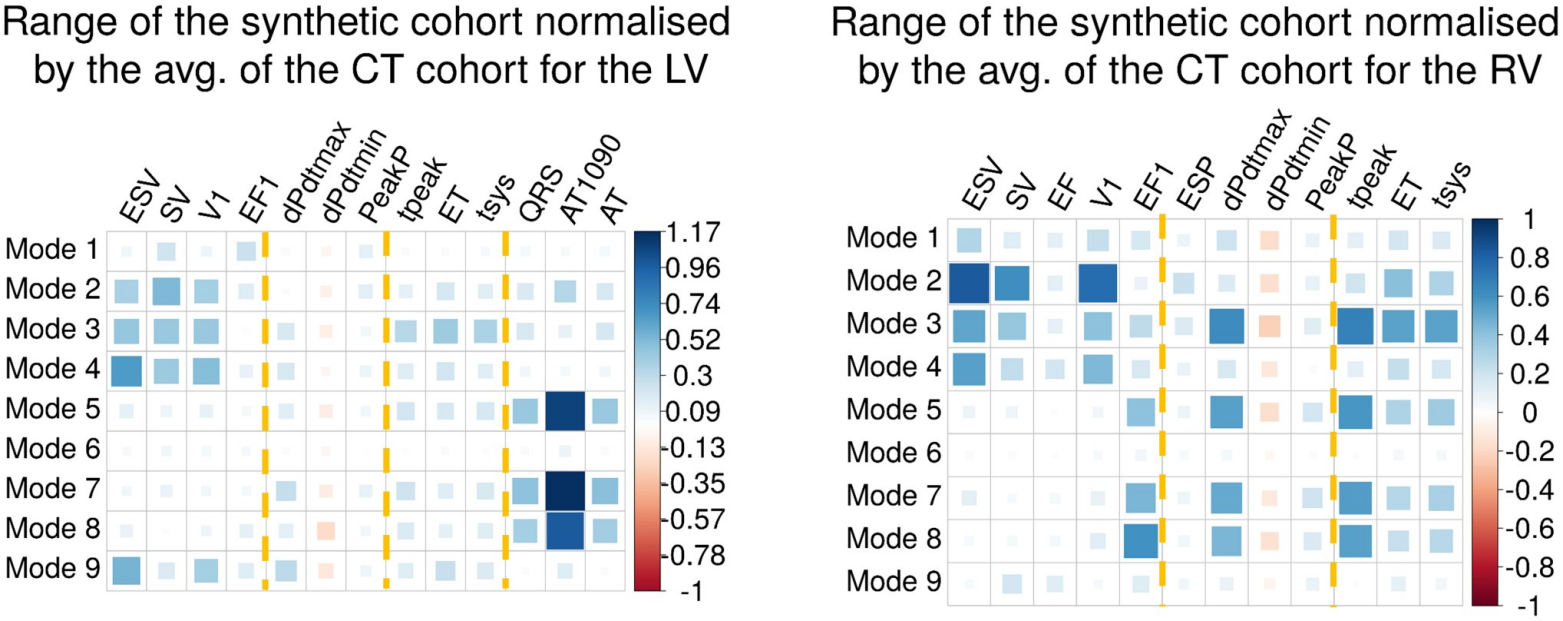
Normalised ranges for the synthetic cohort: For the LV on the left and for the RV on the right. Colour and size indicate the sign and the magnitude of the value. The bigger the value, the more sensitive that phenotype is to changes in that mode. Yellow dotted lines indicate each group of phenotypes: volume-based, pressure-based, time/duration-based and EP-based.

We considered the normalised range of simulation outputs from the synthetic cohort where each mode was adjusted independently (Fig 8). We observe that distinct groups of modes impact groups of different phenotypes. Volume-based phenotypes are more sensitive to changes in modes 2, 3, 4 in both ventricles and 9 in the LV, with the exception of the EF in the RV. Changes in these modes corresponded anatomically in the biggest changes in LV volume. Pressure-based phenotypes are more sensitive to modes 3, 5, 7 and 8 in the RV, being barely sensitive to any modes in the LV. EP-based phenotypes are determined predominantly by modes 5, 7 and 8. This group of modes accounted for a change in sphericity of the heart, and a change in the pulmonary artery diameter. A fourth group of time/duration-based phenotypes are affected by the same modes as the pressure-based phenotypes. For the explanation of each phenotype and its classification, see Table 1.

### 3.7 Global sensitivity analysis

We used GPEs to perform a GSA, using all the modes and training on each phenotype separately. The mean MSE, measured as the squared difference between the predicted and the observed phenotype, of all phenotypes is 10^−4^ (not normalised). The highest MSE occurred for the emulator of the dPdtmin, with a value of 0.001 mmHg^2^/s^2^. The square root of the dPdtmin MSE was 0.032 mmHg/s or 1.66% of the average value.

**Fig 9:**
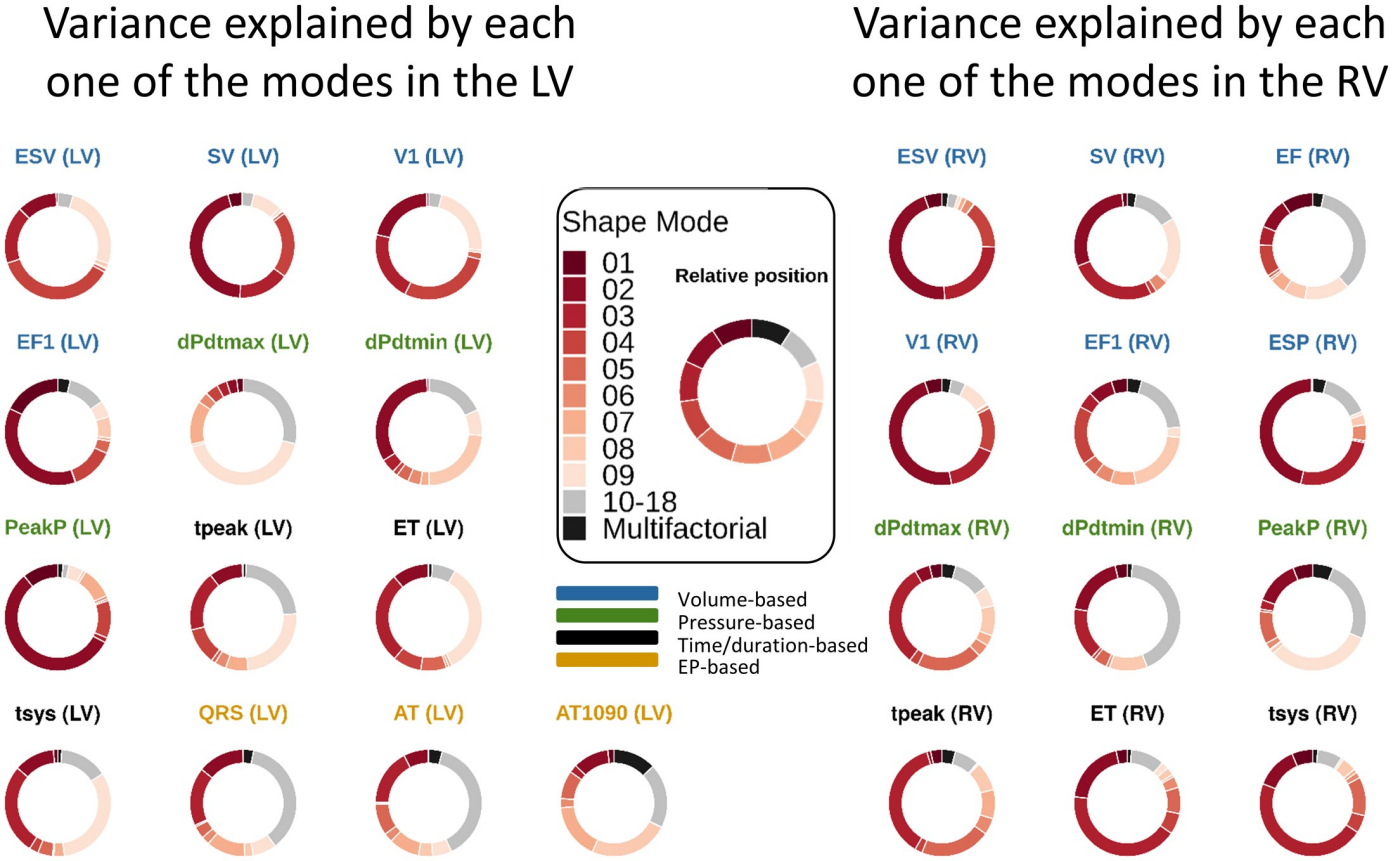
GSA performed using GPEs. A different GP has been used for each one of the phenotype. The bigger the slice in the doughnut chart, the more variance is explained globally by that mode as a first order effect. Interrelations between modes are encompassed in the multifactorial effects. The relative position between modes is consistent across the charts, as indicated in the legend in the centre. The titles of the doughnut charts are colour-coded according to the group of phenotypes they belong to.

We present the GSA results as doughnut charts in Fig 9. Each piece of the the doughnut charts corresponds to the percentage of variance explained globally by that mode, considering only first order effects. The redder the doughnut chart, the more dependent the phenotype is on modes that are important for explaining anatomy; whereas greyer regions represent phenotypes that explain a small amount of anatomical variation. Modes 2, 3 and 9 explain most of the variance in most of the phenotypes. We observe that the volume-based phenotypes in the LV are explained mainly by mode 2 (29.02 ± 15.16%). Mode 9 ranks third in this group of phenotypes, explaining 15.45 ± 9.93% of the variance. This compares with a range of 0.14 ± 0.15% (mode 12) to 24.77 ± 10.26% (mode 4). For the pressure-based phenotypes in the LV, the most relevant modes are mode 2 (31.12 ± 26.82%) and mode 9 (18.24 ± 21.06%). The range in this group of phenotypes for the rest of the modes vary from 0.3 ± 0.32% (mode 12) to 8.58 ± 13.42% (mode 8). Mode 9 explains predominately dPdtmax, tpeak, ET and tsys in the LV, and EF and PeakP in the RV, ranging from 14.07% in the case of the RV EF to 42.46% in the case of dPdtmax in the LV. The EP-based phenotypes can not be explained by a single shape mode.

### 3.8 Local sensitivity analysis in the average mesh

**Fig 10:**
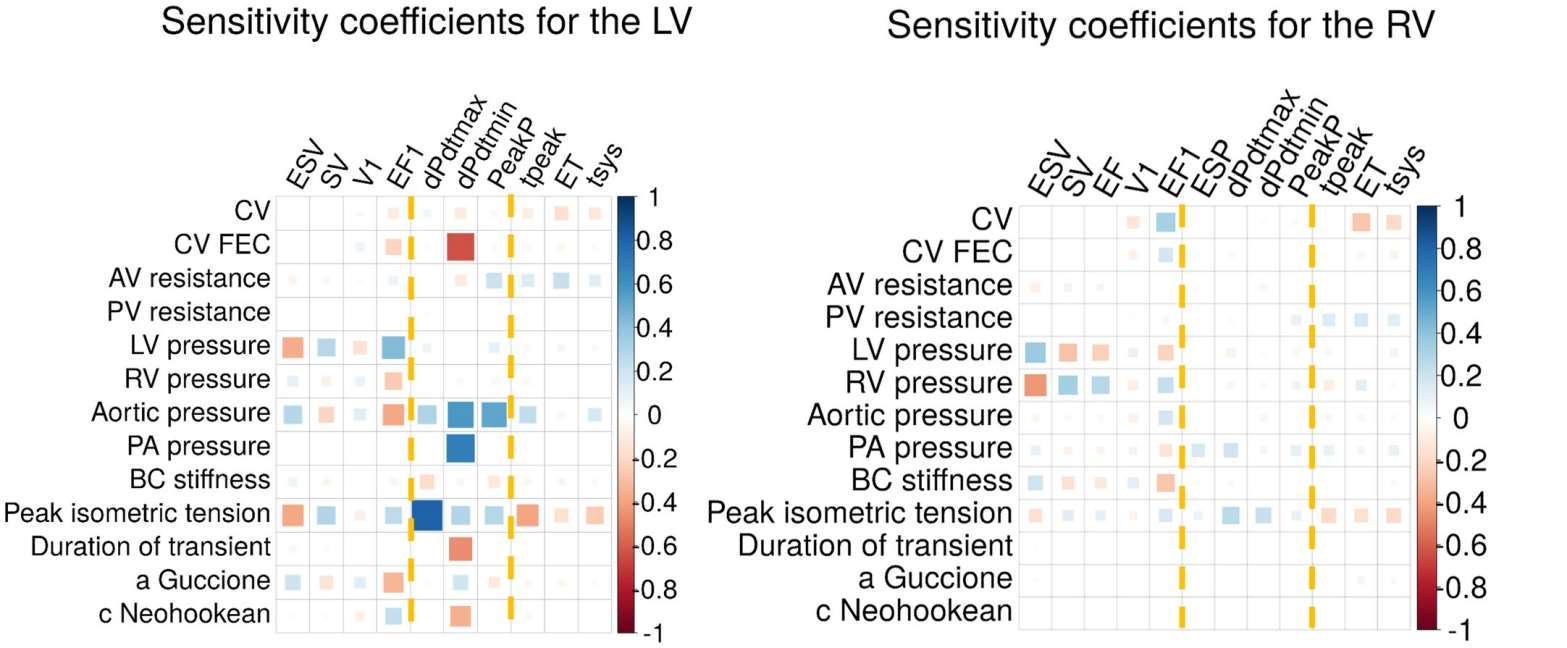
Local sensitivity coefficients for different parameters modified by ± 10% with respect to the baseline value. Each tile indicates percentage of modification of the output per percentage modified of the input. Yellow dotted lines indicate each group of phenotypes: volume-based, pressure-based and time/duration-based.

To quantify the effect that has in our results fixing the parameters, we performed a local sensitivity analysis on the average mesh, shown in Fig 10. Assuming linearity, a value of 1 would indicate that when that parameter is increased by a 10%, the output increases by a 10%. We did not include the EP-based phenotypes in the plot since most of the inputs do not affect the EP activation.

The model parameters with higher effect on LV outputs are peak isometric tension, aortic pressure and LV endocardial pressure with absolute coefficients of 0.32 ± 0.19, 0.29 ± 0.17 and 0.16 ± 0.15, respectively. In the case of the RV, RV endocardial pressure, peak isometric tension and LV endocardial pressure are the parameters with higher effect with absolute coefficients of 0.15 ± 0.14, 0.15 ± 0.06 and 0.12 ± 0.12, respectively. The initial endocardial pressures drive the changes in volume, being the volume-based phenotypes the group mainly affected by alterations in these parameters. The value of peak isometric tension is a key element in triggering the contraction and the force applied in it, affecting mainly the pressure-based phenotypes. Changes in conductivity do not have a noticeable effect except for a change in the CV in the FEC layer, affecting mainly dPdtmin in the LV with a value −0.6. We notice that even if the LV is more sensitive to changes in the input parameters than the RV (absolute values of 0.01 ± 0.2 vs 0.006 ± 0.11), in both cases we consider the sensitivity to be low.

In the case of the EP, the only two input parameters considered affecting its activation are the CV of the myocardium and the CV of the FEC layer. The higher sensitivity is found in the LV total activation time when modifying the CV of the myocardium, with a coefficient of −0.78.

We considered three more scenarios modifying the mesh construction: modified fibre angle, including a fractal tree as model of Purkinje network instead of a FEC layer, and using only the ventricles but with pericardium boundary conditions. In this case, we show the increment of each output with respect to the baseline, normalised by the baseline value. In the case of the wide fibre span in both ventricles (*α*_endo_ = 85°, *α*_epi_ = −65°), we observe the highest absolute changes in EF1 in the RV with a value of 10.51% being the mean absolute effect for all the outputs of 3.22% ± 3%. Analogously, with a narrower fibre span in both ventricles (*α*_endo_ = 75°, *α*_epi_ = −55°) the mean absolute effect is 2.16% ± 1.88% with the highest effect on AT1090 in the LV with a value of −8.29%. Four extra scenarios were analysed, when using the narrow or wide fibre configuration only in the LV or only on the RV. The biggest changes are observe when using the wide configuration in the RV (and the default in the LV), with absolute changes of 3.75 ± 3.16%.

In the case of using a fractal tree as Purkinje fibre network model, the highest absolute changes in EP phenotypes is in AT1090 (LV) as well, with a value of 38.8%. The minimum change is found in QRS duration with a change of 26.16%. Thirdly, when modelling only the ventricles, we find changes of 25.21% ± 18.18% across all phenotypes, with a maximum of 70.36% in dPdtmin in the RV.

## 4 Discussion

In this work we systematically studied the relationship and interplay between anatomical and functional variability in the heart. We created the first virtual cohort of healthy four chamber hearts tested using EM simulations. We showed that shape modes that explain large portions of anatomical variance do not explain equivalent levels of EM functional variation. We demonstrated that there are subtle anatomical changes (e.g. mode 9) that cause a large impact on function. Finally, we found that groups of simulated functional biomarkers (changes in volume, pressure, timing and activation) are dependent on distinct sets of shape modes.

### 4.1 Validation of the reconstructed anatomy and simulated function

Our data consist of anonymised static CT imaging data obtained directly from healthy patients. We do not have access to clinical data such as QRS duration or EF, making a direct validation for each patient unfeasible. Moreover, since we fixed all the parameters for the EM simulations, we could not validate against clinical data (we would need to personalise the models). To validate the cohort, we compared average outcomes from the models and the simulations with published data from cohorts of healthy patients.

We observed an offset of the volume values in our CT cohort, where volumes were smaller (128 ± 24 mL vs 143 ± 34 mL) than those reported from the UK Biobank [51]. This difference seems to be primarily caused by our small sample size (*n* = 20) and by the fact that our cohort is drawn from a different population than UK Biobank. For instance, the UK Biobank population is known to be more health conscious, leaner and non-smokers than the general population [56].

Regarding simulated function, we only personalised the anatomical models. We did not have sufficient data to constrain the mechanics models for these patients. However, we selected material properties and boundary conditions from the literature (S4 Text) and demonstrated that the range of clinically measured simulated function are consistent with reported population values (Subsection 3.3).

### 4.2 The anatomy encoded in a statistical shape model

We found that 9 modes were required to explain 90% of the CT anatomical variance. This is considerably less compared to other whole-heart SSMs, such as the study by Ordas et al. [57] where over 30 modes were required to reach the 90% of variance (or compactness) in a study of 100 cases. However, this sample did include both asymptomatic and coronary artery disease patients, and additional structures such as the descending aorta, which may have increased the anatomical variance. Our results are more in line with the findings by Unberath et al. [58], who required 16 modes to explain 90% of the variability with a sample of 20 “normal male” patients.

We discarded case #20 from the SSM due to the distinct atrial anatomy in that patient. The left PVs branched farther from the atrial body compared to the rest of the cases, creating a more elongated LA. This variability has been observed previously in anatomical studies where, in approximately 23% of the cases, the left atrium had four orifices but with a “short vestibule or funnel-like common vein” [59]. In previous works the authors either did not find this variation in their cases [57, 60] or have excluded the PVs for the atlas creation [61, 62]. Other techniques such as the one reported in [63] could be used to capture the bigger variability of the PVs, since they do not fit in the Gaussian distribution shape density assumed by the SSM.

In previous works on SSM, some cases have been also discarded. In [64], prior to the mesh construction, Mauger et al. excluded up to 85% of the original cases for reasons of age, ethnicity, lack of metadata and cardiac diseases. In [65], Hoogendoorn et al. discarded 4 out of 134 of their cases since several parameters deviated more than 4 SD from the mean.

### 4.3 The simulated functional variability

The cardiac simulations performed were deterministic with common material properties and boundary conditions between cases, therefore any change in the model outputs can only be attributed to changes in the input anatomy.

We focused on phenotypes that were dependent on anatomy and their range was greater than 20% of a baseline mean value. We found that the LV EF, the duration of the isovolumic phases and the LV ESP did not exhibit high variation, fell below this threshold.

The small variation in LV EF is consistent with previous studies [66] where the authors showed that changes in simulated EF were mainly driven by changes in the parameters of the active tension model and the circulatory system model, which we set as fixed parameters in all the cases.

The IRT timing phenortype also show relatively small clinical variability [67]. Nonetheless, there are three possible reasons cardiac cycle timings will have limited variance. Firstly, we simulated a single heart beat with a fixed heart rate, so all timings must be compatible with the cycle length. Secondly, all time-dependent variables in the model are fixed, so the rate of tension development and the duration of contraction are constant and these are likely to drive the timings. And thirdly, our models are coupled to fixed Windkessel models as boundary conditions, as opposed to a closed loop cardiovascular system, where changes in cardiac stroke volume, in either ventricle, can feedback and result in alterations in the preload and afterload that can, in turn, change the cardiac cycle timings. However, previous studies [44] found that boundary conditions did not have a significant influence on cardiac output, albeit in a rat model. Our results from the local sensitivity analysis agree with [44], showing that the results are not overly sensitive to the stiffness of the boundary conditions.

Volume-based phenotypes in both ventricles demonstrate the highest variability. This can potentially be attributed to the variation in the EDV, which comes directly from the reconstruction of the CT images. This high variability in the EDV is likely to lead to higher variability in ESV and SV. Volumetric variability in the model derived form CT images appears to lead to higher variation in simulated volume based indexes of cardiac function.

Finally, the high variability observed in the EP-based phenotypes depends on the area the electrical wave has to cover. The cases in the CT cohort present a high variability in EDV, possibly causing a high variability in ventricular surface area. All this variability can contribute to high variability in the EP-based phenotypes even with the same input conduction velocities.

### 4.4 Anatomico-functional mapping

We found that latter modes can be more important than first modes for explaining simulated function. This is in contrast with earlier LV only studies, which found that the first modes were often the most important for determining strain fields [68]. However, these models only included 6 modes, represented the LV only and did not include atrial or pericardial boundary conditions. This may have led to different contraction patterns and included modes of shape variation more specific to the LV. Based on these findings, future SSMs based only on the LV might not be enough to encode cardiac function.

Different authors have proved in the past that latter modes can potentially lead to a better classification of patient outcomes. Lamata et al. [69] found that mode 14 was an importance risk factor for stratifying pregnant women with hypertension, although they interpreted this fact as possible artefact. In [70], Barbarotta et al. performed a SSM on the LV only, finding that mean strain fields were dependent on latter modes. Shape modes that explain a small amount of anatomical variance can therefore have a far greater impact on simulated phenotypes and this may explain the identification of latter modes as clinical risk markers.

Simulations predicted that sets of phenotypes were explained by common sets of shape modes. Mode 2 and 3 were the principal determinants of LV myocardial mass, while modes 2, 3, 4 and 9 are the main drivers of changes in LV EDV. Increased wall thickness (and hence LV myocardial mass) is associated with increased contraction [71]. Similarly, increased EDV, in the short term, increases contraction [72], however, this effect is countered by the Law of Laplace. The impact of modes 2, 3, 4 and 9 on myocardial mass and EDV, two known determinants of cardiac mechanics, may explain their greater impact on mechanical outputs [71, 72].

We found that the EP-based phenotypes were mainly affected by changes in modes 5, 7 and 8 (Fig 8). When analysed globally in the GSA, this group of phenotypes is explained mainly by mode 10 (16.57 ± 6.26%) and multifactorial interactions (15.67 ± 10.5%). These results compare to a range of 0.37 ± 0.31% (mode 4) to 13.65 ± 4.93% (mode 7) for the remaining modes, being modes 5 and 8 amongst the seven more important modes. Although the importance of modes 5, 7 and 8 is present in both local and global sensitivity analyses, the identification of other modes as important in the GSA might be due to three reasons. Firstly, multifactorial effects were not taken into account in the local sensitivity analysis, since we only modified individual modes separately. Secondly, we modified the modes explaining up to 90% of the accumulative anatomical variance, therefore not checking the effect of modes 10 to 18. And thirdly, the GSA was only performed over the range of modes present in the CT cohort, and therefore not including the extreme cases as in the local sensitivity analysis.

In terms of the pressure-derived phenotypes, dPdtmax is mainly altered by modifications in mode 9, which explains less than 3% of the anatomical variability. This mode determines changes in shape in the atria and in the basal part of the LV septum, creating a bulge right below the aortic outflow tract (see S6 Text). This is in agreement with the literature, where septal thickening has been related to haemodynamic effects [73], and where basal septal hypertrophy is a recognised early sign of adaptation of the heart to hypertension [74].

### 4.5 The impact of fixing model and functional parameters

We performed a local sensitivity analysis modifying ±10% the key parameters of the simulations. In general, we observed a normalised sensitivity of less than 1, the maximum value is of 0.8, or a change in 8% for a change of 10% in the input. This compares with the variability in output with differences in anatomy (Fig 6) where the highest value was in the RV stroke volume, with a range of 124%. This is consistent with normal anatomical variation having a large impact for minor (<10%) variations in material properties. A full global anatomico-functional sensitivity analysis would address this question but the number of simulations to sample this high dimensional space would exceed our available compute capacity.

We included the passive effect of the atria on the ventricle in the reference model. The specific passive properties of the atria did not have a big impact on ventricular function. First, we investigated the impact of a change in end-diastolic pressure, which would normally be informed by atrial contraction. We observed that for a change of 10% in the LV and RV pressure, the outputs change by a 1.6 ± 1.5% and 1.5 ± 1.4%, respectively. Second, we altered atrial passive stiffness to estimate the impact of changes in passive material properties that may be introduced by the presence of an atrial fibre model. In this case, the absolute changes observed for a 10% change in the stiffness are of 0.78% ± 1.22% and 0.03 ± 0.03% in the LV and the RV, respectively. In both cases, the phenotypes showed a low sensitivity to changes in these parameters, consistent with the absence of the atrial features not overly impacting our results.

We also quantified the influence of the choice of the fibre direction with 6 different scenarios. The biggest changes are in the case of the wide configuration (*α*_endo_ = 85° and *α*_epi_ = −65°) in both ventricles. The magnitude of these results suggests that, although the fibre direction is an essential factor in the EM simulations, small changes in their direction do not have a large impact the results.

In the case of using a fractal Purkinje network, we observed a higher sensitivity. In this case, we used the default parameters in terms of density of Purkinje fibres. We did not tune the CV parameters or the density of the fractal tree to match values of the literature as we did with the FEC model. The fractal Purkinje network causes a large, up to 39%, change in EP phenotypes. This difference in results shows that a change of model needs a new tuning of the parameters to achieve physiological behaviour. However, there was limited impact of the fractal Purkinje network on the mechanical phenotypes, with an impact of 7.97% ± 6.89%, ranging from 0.04%*to*21.95%.

The inclusion of the atria on the model allowed us to apply omni-directional spring boundary conditions on the superior veins. We tested the effect of these boundary conditions repeating the simulation on the average mesh with the default parameters but only with the ventricles. As boundary conditions for the simulation, we applied the default pericardial boundary conditions. We found changes of 25.21% ± 18.18% across all phenotypes, with a maximum of 70.36% in dPdtmin in the RV. Analogously to replacing the FEC layer by a fractal Purkinje network, this model would need a new retuning of the parameters to match literature values.

### 4.6 Imaging functionally relevant shape features

The results from the GSA show a low relevance of multifactorial effects (5.18 ± 5.84%) on most of the phenotypes. This compares to a range of no multifactorial effects at all (as in the case of EDV, ESV and V1 in the LV) to a maximum of 27.8% in the case of the AT1090. This means that the interaction between different modes is small compared with the the individual influence of the modes. In terms of clinical relevance, imaging protocols could then focus on detecting changes in individual shape modes that are found to be important for predicting function.

To interpret and visualise each one of the modes, we measured the displacements from the meshes of the extreme3 cohort to the average mesh. The biggest deformations are present in mode 3, with maximum values of approximately 15 mm and mean displacements of 5.8 and 8.8 mm for the LV and the RV, respectively. In mode 9 for instance more subtle changes are present, where the biggest deformations are approximately 6 mm and mean deformations are 1.93 and 1.6 mm for the LV and RV, respectively. These findings suggest that the impact of modes on simulated function might be more dependent on the location than on the magnitude of the changes.

The extreme3 meshes, used to interpret the modes, describe the range covered by over 99% of the expected observations, so we can safely compare with the resolution of the most common imaging protocols. Typical MRI resolution of approximately 1 − 2 [75] mm in-plane would be able to capture changes of mode 9. However, out of plane resolution can be up to 5-10 mm [76]. This means that the ability to determine modes will depend on the orientation of the image with respect to the heart. This problem is augmented in echocardiography since, even if in some cases it can have have submillimetrical resolution [77], the signal-to-noise ratio [78] can limit the ability to make precise anatomical measurements. Cardiac CT would be needed in these cases, where submillimetre resolution is routinely achieved [79].

### 4.7 Limitations

We ran cardiac simulations using models built under certain assumptions and simplifications such as the FEC layer as a Purkinje system model or the ventricles as transversely isotropic materials. We solved the nonlinear equations numerically, which have both accuracy and stability limitations. Our results need to be interpreted considering these factors.

The shape in our models is based on a semi-automatic segmentation. Except for the LV, we built the wall of the other chambers following the shape of the blood pools but fixing the wall thickness (see S1 Text). There may be subtle variations in thickness in the atria and RV that may impact the mechanics in these chambers [19, 80]. We modelled the shape of the valves as closed surfaces since they are used for calculating cavity volumes and for bearing load during when the valves are close. With the endocardium as a fully closed surface, we achieve an evenly distributed applied force, and at the same time, we were able to keep track of volume changes during deformation. In the simplified haemodynamical model, the valves were modelled as diodes with high backward resistance to prevent backflow. The flow through the valves was then computed as the ratio of the pressure drop and the resistance. A more detailed reconstruction of these structures, combined with computational fluid dynamics simulations might lead to more accurate simulation of local pressure dynamics [81].

We modelled organ-scale mechanics using large deformation mechanics solved using the finite element method. Each time step of the simulation requires the solution of a set of nonlinear equations coupled to an ODE boundary condition model. These equations have no guaranteed solution in complex geometries and failure of cardiac mechanics models to reach a converged solution is a known problem [9]. In our study 12% of simulations did not converge, not producing enough data (especially, in specific patient cases in the extreme cohorts) to do the exact same analysis in all the cases. The quality of the meshes, measured with the SJ values (see S5 Text), did not seem to be related to the convergence rate. We noticed that more extreme3 meshes diverged, especially mode 2, potentially reflecting the inability of extreme shape models to converge. While we considered and evaluated different solver conditions and time steps, we did not have the resources, given the ≈ 4000 core-hours per simulation, to ensure every simulation completed. This is likely to prove to be a problem as cardiac mechanics models are applied to larger virtual patient cohorts [20]. Previous studies on larger cohorts used linear mechanics [82], which is more stable but less accurate [83, 84], or focused on LV only meshes [85], which are simpler and more stable anatomies. How best to accommodate simulations that fail to complete remains an open question. Ideally, all simulations would complete and this continues the motivation for advances in numerical methods to improve stability of large deformation mechanics solvers.

A limitation of the SSM is that we do not include all anatomical variation in the LA. Firstly, we restricted the anatomical variability in the topology of the PVs, despite its significant effects on atrial function as [86, 87]. Secondly, we did not include the LA appendage for consistency, it was out of the CT image field of view in some of the cases. The anatomy of the LA appendage has been shown to be a key factor for instance in ischaemic stroke [88] or in the design of occluders to avoid thrombus [89]. To include this in the atlas, a larger cohort with multiple occurrences of variants of left atrial anatomies would be required. With such data, a SSM incorporating the variability in the topology of the LA could be constructed.

In this study we only focused on the impact of anatomy on simulations, despite the importance of fine-tuning and personalisation of parameters for personalised simulations [90]. For instance, increased wall thickness, if paired with myofibre disarray and scarring tissue, can decrease contractility, as in the case of hypertrophic cardiomyopathy [91]. To include these effects, we would also need patient-specific information about the fibre orientation. However, as the dimensionality of the problem increases the number of simulations required to perform a sensitivity analysis also increases. Combined studies of anatomy, boundary conditions and material properties may require a screening process, such as the Morris method [92] to identify a tractable subset of parameters and shape modes that can be studied.

The atria were modelled as a passive neo-hookean material. Although it is a significant simplification, simulations of atrial mechanics are not as widespread as ventricular mechanics. A more thorough investigation on atrial modelling and simulation for four-chamber cardiac meshes is out of the scope of this paper. Nevertheless, we added them to obtain more physiological boundary conditions on the pulmonary veins and venae cavae.

## 5 Publicly accessible virtual cohort

We have made all the meshes from the CT and synthetic cohort available for the community in .vtk format available on 10.5281/zenodo.4590294 and 10.5281/zenodo.4593739. We have added 1000 more meshes modifying the PCA weights randomly withing 2 SD range, available in 10.5281/zenodo.4506930. The same anatomical structures are present in all the meshes described, but fibres and UVC were not included in the extra 1000 batch.

A VTK file for each mesh was included (in ASCII) as an UNSTRUCTURED GRID. In all the cases the following fields were included: POINTS, with the coordinates of the points in mm; CELL_TYPES, having all of the points the value 10 since they are tetrahedra; CELLS, with the indices of the vertices of every element; and CELL_DATA corresponding to the meshing tags.

In the case of the CT and synthetic cohorts some extra fields were added: two VECTORS fields, with the fibres and sheet directions (one fibre and sheet direction per element) and a POINT_DATA field with four LOOKUP_TABLE subfield corresponding to the UVC in the order *ρ*, *ϕ*, *Z* and *V*. Since the UVC were defined only in the ventricles, a value of −10 was established for all the coordinates in the elements not belonging to the ventricular myocardium.

For every cohort, a file with the value of the weights as well as the functional phenotypes has been included for each heart.

## 6 Conclusion

Our results showed that using only the main modes of shape we may not capture sufficient anatomical variability to perform accurate functional cardiac simulations. Conversely, these simulations can map smaller shape modes, that may be found irrelevant in standard shape analysis, to meaningful changes in cardiac function. Moreover, the mechanical, electrical, and pressure-derived phenotypes are dependent on different subsets of anatomical modes. These results suggest that tailored imaging protocols should be used for creating models of specific cardiac function.

## Supporting information

S1 Text Detailed methodology of the pipeline followed to build the meshes of the CT cohort, from segmentation to fibre assignment. (PDF)

S2 Text Details on the Universal Ventricular Coordinates (UVC). (PDF)

S3 Text Detailed methodology of the pipeline followed to build the meshes of the synthetic cohort). (PDF)

S4 Text Details on the electro-mechanical simulations, including the values given to the material parameters. (PDF)

S5 Text Details on the scaled jacobian (SJ) quality control metric, and its value on the meshes. (PDF)

S6 Text Detailed possible interpretation of mode 9. (PDF)
