# Correction: Linking statistical shape models and simulated function in the healthy adult human heart

**DOI:** 10.1371/journal.pcbi.1010196

**Published:** 2022-05-25

**Authors:** Cristobal Rodero, Marina Strocchi, Maciej Marciniak, Stefano Longobardi, John Whitaker, Mark D. O’Neill, Karli Gillette, Christoph Augustin, Gernot Plank, Edward J. Vigmond, Pablo Lamata, Steven A. Niederer

The funding statement for this article should read as follows:

“CR and MM have received funding from the European Union’s Horizon 2020 Research and innovation programme under the Marie Sklodowska-Curie Grant Agreement No 764738. MS was supported by an unrestricted Abbott educational grant through the Centre for Doctoral Training in Medical Imaging at King’s College London. CA received support from the Marie Sklodowska-Curie fellowship (https://ec.europa.eu/research/mariecurieactions/), grant number 750835. GP received support from the Austrian Science Fund (FWF) (https://fwf.ac.at/en/); grant number PI2760-B30. PL holds a Wellcome Trust Senior Research Fellowship (209450/Z/17/Z) and is supported by BHF [PG/16/75/32383]. SAN is supported by NIH R01-HL152256, ERC PREDICT-HF 453 (864055), BHF (RG/20/4/34803), EPSRC (EP/P01268X/1). This work was supported by the Wellcome/EPSRC Centre for Medical Engineering [WT 203148/Z/16/Z]. This work made use of ARCHER (http://www.archer.ac.uk/), the UK national high-performance computing service located at the University of Edinburgh and funded by the Office of Science and Technology through Engineering and Physical Sciences Research Councils High-End Computing Programme. The funders had no role in study design, data collection and analysis, decision to publish, or preparation of the manuscript.”
