# Supplementary material for: Linking statistical shape models and simulated function in the healthy adult human heart
Source: PLoS Comput Biol. 2021 Apr 15;17(4):e1008851. doi: 10.1371/journal.pcbi.1008851 (PMC8049237; doi:10.1371/journal.pcbi.1008851)
Supplement: S1 Text — (PDF) [file pcbi.1008851.s001.pdf]

S1 Details on the CT cohort pipeline

S1.1 Segmentation

We performed semi-automatic segmentation of the CT images. We initialised the segmentation using a tool based on automated marginal space learning and steerable features [1]. The segmentation labels were automatically created for the left ventricular (LV) blood pool, papillary muscles and trabeculations, LV myocardium, right ventricular (RV) blood pool, left atrial (LA) blood pool, right atrial (RA) blood pool and aortic blood pool.

We augmented these labels using the open source image processing software Seg3D [2]. We set the thickness to 2 mm for the atria [3, 4], 3.5 mm for the RV [5], 2 mm for the aorta [6] and 3.5 mm for the pulmonary artery outflow tract, see Fig A.

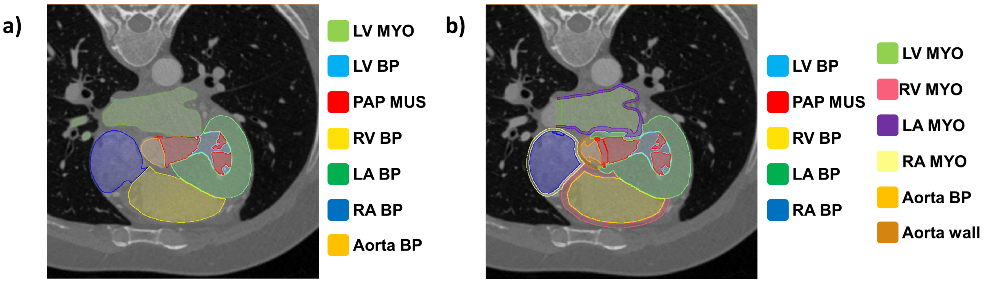

Fig A. a): The result of the automatic segmentation of the blood pools and LV myocardium. b): Augmentation of the segmentation with the labels mentioned in the text. BP stands for blood pool, MYO for myocardium and PAP MUS for papillary muscles and trabeculations.

We clipped the PVs and the venae cavae close to the atrial walls. In some of the cases the LA appendage was not fully captured in the CT image. To maintain topological consistency, we discarded the LA appendage in all models. We preserved small parts of the vessels for realistic mechanics boundary conditions as in [7] (Fig B, labels 18 to 24). To compute the volume of each chamber we closed the chambers adding extra surfaces at the vessels locations. We created the structures corresponding to the valves by dilating the adjacent blood pools one into the other with an approximate thickness of 2 mm, to ensure that the meshing was performed with no holes through the valve plane layer. The 31 created labels are shown and listed in Fig B.

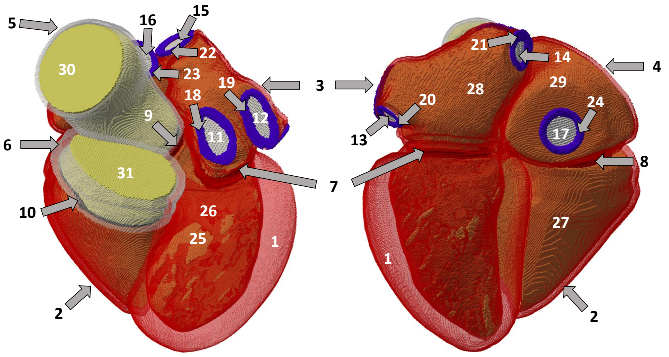

1	LV myocardium	9	Aortic valve	17	Inferior vena cava inlet	25	LV blood pool
2	RV myocardium	10	Pulmonary valve	18	LA appendage border	26	LV trabeculae
3	LA myocardium	11	LA appendage inlet	19	Right inferior PV border	27	RV blood pool
4	RA myocardium	12	Left superior PV inlet	20	Left inferior PV border	28	LA blood pool
5	Aorta	13	Left inferior PV inlet	21	Left superior PV border	29	RA blood pool
6	Pulmonary artery	14	Right inferior PV inlet	22	Right superior PV border	30	Aorta cavity
7	Mitral valve	15	Right superior PV inlet	23	Superior vena cava border	31	Pulmonary artery cavity
8	Tricuspid valve	16	Superior vena cava inlet	24	Inferior vena cava border		

Fig B. Segmentation of case #02 before the smoothing step with the 31 consistent labels. The labels with red identification numbers mark the elements remaining in the final meshes. PV stands for the pulmonary vein.

We smoothed and upsampled each segmentation to obtain an isotropic voxelisation of 0.15 mm [8]. This step reduced the 'staircase' effects in the constructed meshes, especially at the element borders [9].

S1.2 Mesh creation

We built unstructured tetrahedral meshes of all the segmented tags except for blood pools and papillary muscles and trabeculae, using the Computational Geometry Algorithm Library (CGAL) [10] with average edge length of 1 mm. The produced mesh before any further modification can be seen in Figure C.

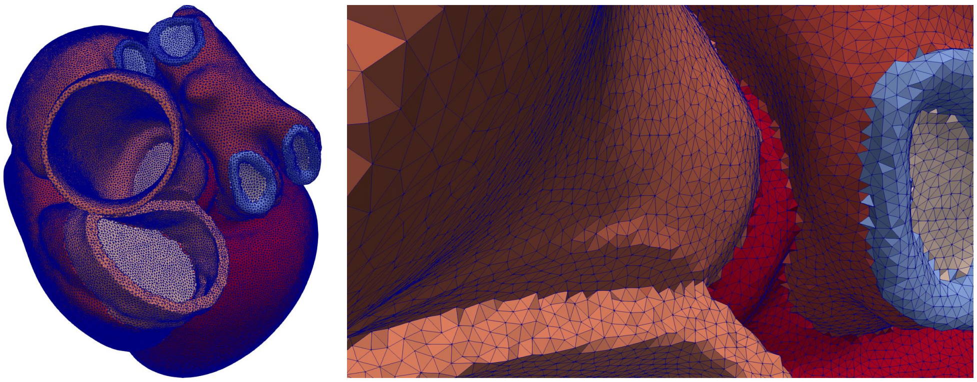

Fig C. The mesh built from the images of case #02. The average edge length of the mesh is 1 mm. The blood pools and the papillary muscles labels were disregarded. The mesh elements (chambers, vessels and borders) were labelled with different tags.

To run EM simulations we needed to extract the endocardium of the four chambers and close them with the different valve and/or veins planes. This was achieved using the Meshtool software [11] by firstly extracting all the surfaces, separating by connected components and merging the surfaces corresponding to the endocardia and the valves/veins planes.

S1.3 Fibre assignment

We assigned a fibre field to the meshes [12]. This represents the preferential cell orientation and provides a local coordinate system for describing material properties including active contraction and conductivity that align with tissue micro structure. We set the transmural myofibre direction to vary linearly from the endocardium to the epicardium, from 80° to −60° [13, 14] respectively. Besides the main fibre direction, we also consider the sheetlets where the fibres are organised. The orientation of the sheetlets is perpendicular to the longitudinal direction, and this angle (called here as transverse angle) can be modified across the myocardial wall. In our simulations we assumed transversely isotropic material, therefor we did not use the sheetlet change of directions. For completeness, we provide the meshes with the sheetlets directions included. We set the transverse angle to vary linearly from −65° in the endocardium to 25° in the epicardium [15], see Fig D.

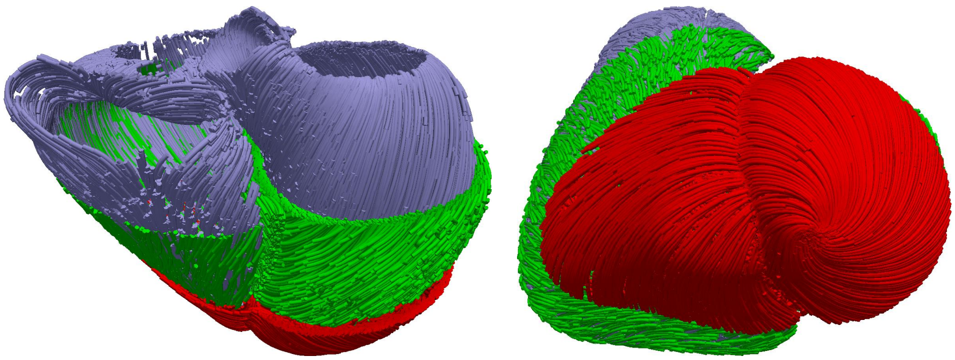

Fig D. The orientation of myofibres assigned to the cells in the ventricles, based on [12–15].

References

1. Zheng Y, Barbu A, Georgescu B, Scheuering M, Comaniciu D. Four-Chamber Heart Modeling and Automatic Segmentation for 3-D Cardiac CT Volumes Using Marginal Space Learning and Steerable Features. IEEE Transactions on Medical Imaging. 2008;27(11):1668–1681. doi:10.1109/TMI.2008.2004421.

2. CIBC. Seg3D: Volumetric Image Segmentation and Visualization.; 2016. Available from: `http://www.sci.utah.edu/software/seg3d.html`.

3. Beinart R, Abbara S, Blum A, Ferencik M, Heist K, Ruskin J, et al. Left Atrial Wall Thickness Variability Measured by CT Scans in Patients Undergoing Pulmonary Vein Isolation. Journal of Cardiovascular Electrophysiology. 2011;22(11):1232–1236. doi:10.1111/j.1540-8167.2011.02100.x.

4. Varela M, Kolbitsch C, Theron A, Morgan R, Henningsson M, Schaeffter T, et al. 3D high-resolution atrial wall thickness maps using black-blood PSIR. Journal of

Cardiovascular Magnetic Resonance. 2015;17(S1):P239. doi:10.1186/1532-429X-17-S1-P239.

5. Ho SY, Nihoyannopoulos P. Anatomy, echocardiography, and normal right ventricular dimensions. Heart (British Cardiac Society). 2006;92 Suppl 1(Suppl 1):2–13. doi:10.1136/hrt.2005.077875.

6. Mensel B, Kühn JP, Schneider T, Quadrat A, Hegenscheid K. Mean Thoracic Aortic Wall Thickness Determination by Cine MRI with Steady-State Free Precession. Academic Radiology. 2013;20(8):1004–1008. doi:10.1016/j.acra.2013.03.014.

7. Strocchi M, Augustin CM, Gsell MA, Karabelas E, Neic A, Gillette K, et al. A publicly available virtual cohort of four-chamber heart meshes for cardiac electro-mechanics simulations. PLoS One. 2020;15(6):e0235145.

8. Knoll F, Bredies K, Pock T, Stollberger R. Second order total generalized variation (TGV) for MRI. Magnetic Resonance in Medicine. 2011;65(2):480–491. doi:10.1002/mrm.22595.

9. Crozier A, Augustin CM, Neic A, Prassl AJ, Holler M, Fastl TE, et al. Image-Based Personalization of Cardiac Anatomy for Coupled Electromechanical Modeling. Annals of biomedical engineering. 2016;44(1):58–70. doi:10.1007/s10439-015-1474-5.

10. The CGAL Project. CGAL User and Reference Manual. 4th ed. CGAL Editorial Board; 2017. Available from: `http://doc.cgal.org/4.11/Manual/packages.html`.

11. Neic A, Gsell MAF, Karabelas E, Prassl AJ, Plank G. Automating image-based mesh generation and manipulation tasks in cardiac modeling workflows using Meshtool. SofwareX. 2020;11. doi:10.1016/j.softx.2020.100454.

12. Bayer JD, Blake RC, Plank G, Trayanova NA. A Novel Rule-Based Algorithm for Assigning Myocardial Fiber Orientation to Computational Heart Models. Annals of Biomedical Engineering. 2012;40(10):2243–2254. doi:10.1007/s10439-012-0593-5.

13. Land S, Park-Holohan SJ, Smith NP, dos Remedios CG, Kentish JC, Niederer SA. A model of cardiac contraction based on novel measurements of tension development in human cardiomyocytes. Journal of Molecular and Cellular Cardiology. 2017;106:68–83. doi:10.1016/j.yjmcc.2017.03.008.

14. Land S, Niederer SA. Influence of atrial contraction dynamics on cardiac function. International Journal for Numerical Methods in Biomedical Engineering. 2018;34(3):e2931. doi:10.1002/cnm.2931.

15. Streeter DD, Spotnitz HM, Patel DP, Ross J, Sonnenblick EH. Fiber orientation in the canine left ventricle during diastole and systole. Circulation research. 1969;24(3):339–47. doi:10.1161/01.RES.24.3.339.
