# Supplementary material for: Linking statistical shape models and simulated function in the healthy adult human heart
Source: PLoS Comput Biol. 2021 Apr 15;17(4):e1008851. doi: 10.1371/journal.pcbi.1008851 (PMC8049237; doi:10.1371/journal.pcbi.1008851)
Supplement: S2 Text — (PDF) [file pcbi.1008851.s002.pdf]

S2 Universal Ventricular Coordinates

We calculated UVC on the CT and synthetic cohorts, following the approach presented in [1]. The UVC consist of four different numbers for each point in the ventricular myocardium: ρ, corresponding to the transmural coordinate, with $\rho = 0$ at the endocardium and $\rho = 1$ at the epicardium; ϕ, corresponding to the rotational coordinate, with $\phi = \pm\pi$ at the LV free wall, negative values in the posterior part of the heart and positive values in the anterior part of the heart; Z, corresponding to the apicobasal coordinate, with $Z = 0$ at the apical junction of the ventricles and $Z = 1$ at the base of the heart; and V, corresponding to the transventricular coordinate, with $V = -1$ if the point belongs to the LV, and $V = 1$ if it belongs to the RV. An example of the UVC is shown in Fig A.

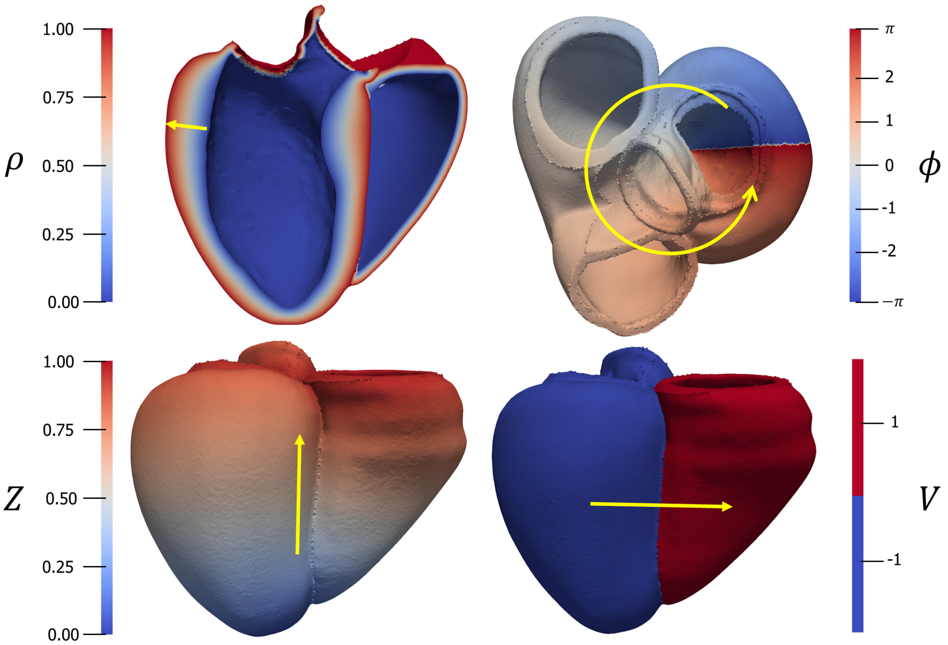

Fig A. Visualisation of the UVC in a single mesh. All coordinates are continuous except for V which takes two discrete values. The yellow arrows indicate the increasing value of the coordinate. ρ corresponds to the transmural coordinate, ϕ to the rotational coordinate, Z to the apicobasal coordinate and V to the transventricular coordinate.

References

1. Bayer J, Prassl AJ, Pashaei A, Gomez JF, Frontera A, Neic A, et al. Universal ventricular coordinates: A generic framework for describing position within the heart and transferring data. Medical Image Analysis. 2018;45:83–93. doi:10.1016/j.media.2018.01.005.
