# Supplementary material for: Linking statistical shape models and simulated function in the healthy adult human heart
Source: PLoS Comput Biol. 2021 Apr 15;17(4):e1008851. doi: 10.1371/journal.pcbi.1008851 (PMC8049237; doi:10.1371/journal.pcbi.1008851)
Supplement: S3 Text — (PDF) [file pcbi.1008851.s003.pdf]

S3 Details on the synthetic cohort pipeline

We created a SSM from the CT cohort meshes. We created a pipeline ending with viable meshes for EM simulations.

To create the SSM, we required every subject to be in the same physical space (i.e., to be rigidly aligned). First, we translated the 19 meshes so the middle point between the mitral valve barycentre (MVB) and tricuspid valve barycentre (TVB) was in the same spatial coordinates. We computed the most apical point in the RV septum (p). We chose an arbitrary reference model and aligned the corresponding points MVB, TVB and p of each model to those of the reference model. Note that we did not scaled the models as is done in a Procrustes transformation such as in [1].

To avoid point-to-point correspondence, we chose to represent the surfaces as currents [2] and embed them in a set of a regular grid of landmark points.

We computed an anatomical average shape (or atlas) using the Deformetrica software and defined the shape variability with respect to the average shape [3, 4]. For each heart, we extract its surface and represent it as deRham currents. We can approximate the current of a triangulated surface by the set of points at the triangles barycentres and the vectors with origin these centres and oriented along the triangle normals (Dirac delta currents) [4].

Using the the Large Deformation Diffeomorphic Metric Mapping [2], the 3D shape representations are compared through diffeomorphic transformations with the 3D space. We set a representative mesh as an initial template and the identity function as the initial transformation. For each shape, by applying a gradient descent algorithm, we update the template and the deformations to minimise the distance between the deformed template and the given shape as defined in Eq (13) in [5]. The distances between currents are defined as in [3], involving a Gaussian kernel.

The deformation functions depend on a set of uniformly distributed control points in which the shapes are embedded, and the deformation vectors attached to these points. In contrast to deforming the mesh in a pointwise manner, this method deforms the whole space where the meshes are, through the control points, see Fig A.

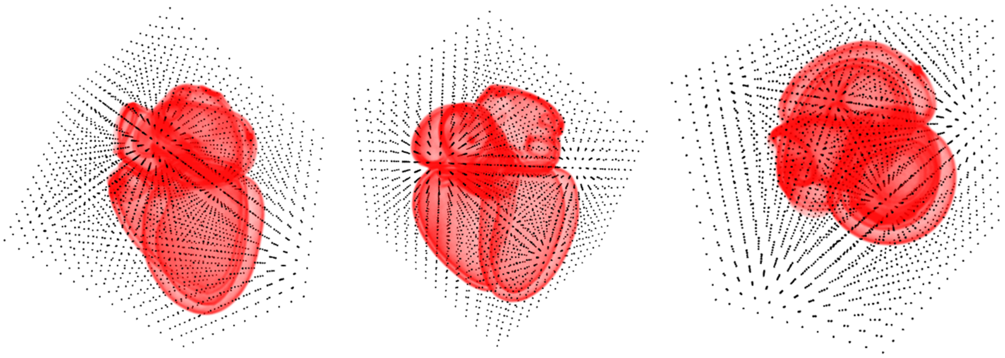

Fig A. Lateral, superior and basal view of the template mesh (represented as currents) with the control points.

We performed a singular value decomposition on the matrix formed by the deformation vectors defined as the difference between the control points of the average mesh and the control points of each one of the meshes. This method provides us with orthogonal bases or modes (analogous to eigenvectors) and variability explained by each mode encoded in the corresponding eigenvalues.

The result is that any patients' heart can be described by the average mesh plus the sum of the modes multiplied by the corresponding weights for a given patient. The method provides a common set of modes (changes in shape) that can be used to describe all cases and they reduce the complex shape with thousands of vertices down to a limited set of scalar values that can encode each patient's heart.

The number of components chosen to represent the data set expresses the trade-off between model complexity and the amount of explained variability. The components are the eigenvectors associated with the covariance matrix (constructed based on the data set):

$$\Sigma = \frac{1}{N}\sum_{i=1}^{N}\mathbf{x}_i\mathbf{x}_i^T, \tag{1}$$

with N denoting the size of the data set and x being the samples. The greater the eigenvalue associated to certain eigenvector, the more information is enclosed within the vector. When normalised, eigenvalues denote the percentages of explained variability.

We transformed all the surface meshes to volumetric meshes to allow for finite element simulations. The transformation was performed with parameters reflecting the quality of the CT cohort meshes. Finally, we labelled the separate elements of the meshes and merged them into single models.

We performed the rigid transformation of the meshes, surface extraction, splitting the mesh into elements, labelling and merging using the VTK package [6] in Python. We registered the meshes with Deformetrica software [7] and its atlas construction module. We transformed from surface to volumetric meshes with gmsh [8].

References

1. Zhou SK, Xu D. A probabilistic framework for multiple organ segmentation using learning methods and level sets. In: Medical Image Recognition, Segmentation and Parsing. Elsevier; 2016. p. 157–178.

2. Vaillant M, Glaunès J. Surface matching via currents. In: Biennial International Conference on Information Processing in Medical Imaging. Springer; 2005. p. 381–392.

3. Bône A, Colliot O, Durrleman S. Learning distributions of shape trajectories from longitudinal datasets: a hierarchical model on a manifold of diffeomorphisms. In: Proceedings of the IEEE Conference on Computer Vision and Pattern Recognition; 2018. p. 9271–9280.

4. Mansi T, Voigt I, Leonardi B, Pennec X, Durrleman S, Sermesant M, et al. A statistical model for quantification and prediction of cardiac remodelling: Application to tetralogy of fallot. IEEE transactions on medical imaging. 2011;30(9):1605–1616.

5. Durrleman S, Pennec X, Trouvé A, Ayache N. Statistical models of sets of curves and surfaces based on currents. Medical image analysis. 2009;13(5):793–808.

6. Community V. VTK: an open-source toolkit for 3D computer graphics, image processing, and visualization; 2006–. Available from: `https://pypi.org/project/vtk/`.

7. Durrleman S, Prastawa M, Charon N, Korenberg JR, Joshi S, Gerig G, et al. Morphometry of anatomical shape complexes with dense deformations and sparse parameters. NeuroImage. 2014;101:35–49.

8. Geuzaine C, Remacle JF. Gmsh: A 3-D finite element mesh generator with built-in pre-and post-processing facilities. International journal for numerical methods in engineering. 2009;79(11):1309–1331.
