# Supplementary material for: Linking statistical shape models and simulated function in the healthy adult human heart
Source: PLoS Comput Biol. 2021 Apr 15;17(4):e1008851. doi: 10.1371/journal.pcbi.1008851 (PMC8049237; doi:10.1371/journal.pcbi.1008851)
Supplement: S4 Text — (PDF) [file pcbi.1008851.s004.pdf]

S4 Details on the electro-mechanical simulations

For the electro-mechanical simulations we followed a similar pipeline as in [1]. We ran EP simulations with the reaction-eikonal model [2], in which the depolarisation is ruled by the eikonal equation:

$$\|\nabla t_a\|_{\mathbf{V}} = 1, \tag{1}$$

where t_a is the wavefront arrival time and $\mathbf{V}$ is the (symmetric positive definite) tensor containing the squared wave velocities associated to the fibre ($\mathbf{f}_0$), sheet ($\mathbf{f}_0$) and sheet normal ($\mathbf{n}_0$) directions.

As an initial condition, we stimulated points in the endocardium of the mesh, using the UVC. We set the constraint on parameter $Z \leq 0.33$, to limit the stimulation to the bottom part of the ventricles simulating the outbreak of the His-Purkinje network terminals. To simulate the early endocardial activation [3], a fast endocardial conduction (FEC) layer was labelled in the meshes, where we set up a higher conduction velocity. The layer covered the 70% of the bottom apicobasal length ($Z \leq 0.7$ in UVC) and is 1 element thick in the endocardium. These parameters are related to the most dense part of the Purkinje network [4]. The only conductive tissue was the ventricular myocardium - activation was not simulated in the atria. See table A for the values of all the EP parameters. Details on the solver and numerical scheme for the EP have been published previously elsewhere [2]. The solver has also been validated in N-version benchmark studies [5].

Parameter	Value (reference)
CV (fibre)	0.8 m/s [6]
CV (transfibre)	0.23 m/s [7]
CV (FEC)	5.6 m/s [8]
Depolarisation threshold	−60 mV [9]

Table A. Parameter values for the reaction-eikonal model (used only in the ventricles). CV stands for conduction velocity and FEC for fast endocardial conduction layer.

We simulated the large deformation mechanics in a Lagrangian reference system [10]. To simulate stresses in the reference configuration we used the model

$$\mathbf{S}_{\text{pas}} = 2\frac{\partial \Psi(\mathbf{C})}{\partial \mathbf{C}} \tag{2}$$

where $\mathbf{S}_{\text{pas}}$ is the passive component of the second Piola-Kirchhoff stress tensor and $\mathbf{C}$ is the right Cauchy-Green deformation tensor.

The ventricular myocardium was modelled as a hyperelastic transversely isotropic material with Guccione's strain energy function [11]:

$$\boldsymbol{\Psi}(\mathbf{C}) = \frac{\kappa}{2}(\ln J)^2 + \frac{a}{2}\left(e^Q - 1\right), \tag{3}$$

$$Q = b_f E_{ff}^2 + b_t\left(E_{ss}^2 + E_{nn}^2 + 2E_{sn}^2\right) + 2b_{fs}\left(E_{fs}^2 + E_{fn}^2\right), \tag{4}$$

with

$$E_{ij} = \mathbf{i}_0 \cdot \overline{\mathbf{E}}\mathbf{j}_0, \tag{5}$$

$$\overline{\mathbf{E}} = \frac{1}{2}\left(\overline{\mathbf{C}} - \mathbf{I}\right), \tag{6}$$

$$\overline{\mathbf{C}} = J^{-2/3}\mathbf{C} \tag{7}$$

being $\overline{\mathbf{E}}$ the modified isochoric Green-Lagrange strain tensor and J the determinant of the Jacobian matrix of the deformation gradient tensor, for $\mathbf{i}, \mathbf{j} = \mathbf{f}, \mathbf{s}, \mathbf{n}$. See table B for the values of the parameters of this constitutive law.

**Parameter**	**Value**
a	1.7 kPa
b_f	8
b_{fs}	4
b_t	3
κ	1000 kPa

Table B. Parameter values for the Guccione's law in the ventricle, extracted from [12].

The remaining tissues were modelled as non-contracting neo-Hookean materials [13] following the strain energy function [1]:

$$\mathbf{\Psi}(\mathbf{C}) = \frac{\kappa}{2}(J-1)^2 + \frac{c}{2}(\mathrm{tr}(\overline{\mathbf{C}}) - 3), \tag{8}$$

where tr is the trace and c and κ the material parameters. The values of κ and c for the valves was set to 1000 kPa (as an arbitrary high value) to enforce material incompressibility. See table C for the values of all the parameters of this constitutive law.

**Parameter**	**Value [reference]**
κ	1000 kPa
c_{atria}	7.45 kPa [14]*
c_{veins}	7.45 kPa [14]*
c_{aorta}	26.66 kPa [15]*
c_{PA}	3.7 kPa [16]*
c_{valves}	1000 kPa

Table C. Parameter values for the Neo-Hookean material model used in the atria and veins. * extracted as average values of the reference cited.

To simulate the active stress of the ventricles triggered by electrical activation, we used the phenomenological activation-based Tanh Stress model [17, 18]. See table D for the values of the parameters of this constitutive law. Details on the solver and numerical scheme for the EM have been published previously elsewhere [19]. The solver has also been validated in N-version benchmark studies [20].

**Parameter**	**Value)**
EM delay	20 ms
Peak isometric tension	120 kPa
Time constant relaxation	50 ms
Time constant contraction	50 ms
Duration of transient	550 ms

Table D. Parameter values for the Tanh strees active tension model (only applied to the ventricles). All the values were hand-tuned.

The preload of the ventricles was modelled via constant atrial pressures, while the afterload was modelled with two three-element Windkessel models, one for the systemic circulation and one for the pulmonary circulation [21]. For the values of the initial pressures see table E.

Parameter	Value (reference)
LV endocardial pressure	1.6 kPa [22]
RV endocardial pressure	0.8 kPa [23]
Aortic pressure	77 mmHg [24]
Pulmonary artery pressure	17.4 mmHg [25]

Table E. Parameter values for initial pressures.

The value of the backward resistances for all the valves were set to either 1000 or 10000 mmHg/mL/s as arbitrary high values to avoid backflow or regurgitation. The forward resistances of the valves have been set to 0 mmHg/mL/s for the aorta and pulmonary artery to achieve numerical convergence and an EF in a healthy range. In the case of the mitral and tricuspid valves, a value of 0.05 mmHg/mL/s was set to allow for physiological filling. Similar values were also used in [26].

Parameter	Value [reference]
Aortic valve: serial resistor	0.03 mmHg s/mL [27]
Aortic valve: parallel resistor	0.63 mmHg s/mL [27]
Aortic valve: capacitor	5.16 mL/mmHg [27]
Mitral valve peak forward flow resistance	0.05 mmHg/mL/s
Mitral valve peak backward flow resistance	1000 mmHg/mL/s
Aortic valve peak forward flow resistance	0 mmHg/mL/s
Aortic valve peak backward flow resistance	10000 mmHg/mL/s
Pulmonary valve: serial resistor	0.015 mmHg s/mL [28]
Pulmonary valve: parallel resistor	0.1008 mmHg s/mL [28]
Pulmonary valve: capacitor	21.156 mL/mmHg [28]
Tricuspid valve peak forward flow resistance	0.05 mmHg/mL/s
Tricuspid valve peak backward flow resistance	1000 mmHg/mL/s
Pulmonary valve peak forward resistance	0 mmHg/mL/s
Pulmonary valve peak backward resistance	10000 mmHg/mL/s

Table F. Parameter values for the 3-elements Windkessel model used for the circulatory system (and valves).

The right pulmonary veins and the superior vena cava were assigned omni-directional spring boundary conditions, to allow spring-like motion within them [29, 30]. To achieve a physiologically plausible motion with downward motion of the atrioventricular plane and limited motion of the apex, we simulated the pericardium applying springs (Robin boundary conditions) normally to the epicardial surface [26].

References

1. Marx L, Gsell MA, Rund A, Caforio F, Prassl AJ, Toth-Gayor G, et al. Personalization of electro-mechanical models of the pressure-overloaded left ventricle: fitting of Windkessel-type afterload models. Philosophical Transactions of the Royal Society A. 2020;378(2173):20190342.

2. Neic A, Campos FO, Prassl AJ, Niederer SA, Bishop MJ, Vigmond EJ, et al. Efficient computation of electrograms and ECGs in human whole heart simulations using a reaction-eikonal model. Journal of Computational Physics. 2017;346:191–211. doi:10.1016/J.JCP.2017.06.020.

3. Hyde ER, Behar JM, Claridge S, Jackson T, Lee AWC, Remme EW, et al. Beneficial Effect on Cardiac Resynchronization From Left Ventricular

Endocardial Pacing Is Mediated by Early Access to High Conduction Velocity Tissue. Circulation: Arrhythmia and Electrophysiology. 2015;8(5):1164–1172. doi:10.1161/CIRCEP.115.002677.

4. Vigmond EJ, Clements C. Construction of a Computer Model to Investigate Sawtooth Effects in the Purkinje System. IEEE Transactions on Biomedical Engineering. 2007;54(3):389–399. doi:10.1109/TBME.2006.888817.

5. Niederer SA, Kerfoot E, Benson AP, Bernabeu MO, Bernus O, Bradley C, et al. Verification of cardiac tissue electrophysiology simulators using an N-version benchmark. Philosophical Transactions of the Royal Society A: Mathematical, Physical and Engineering Sciences. 2011;369(1954):4331–4351.

6. Nanthakumar K, Jalife J, Massé S, Downar E, Pop M, Asta J, et al. Optical mapping of Langendorff-perfused human hearts: establishing a model for the study of ventricular fibrillation in humans. American Journal of Physiology-Heart and Circulatory Physiology. 2007;293(1):H875–H880.

7. Roberts DE, Hersh LT, Scher AM. Influence of cardiac fiber orientation on wavefront voltage, conduction velocity, and tissue resistivity in the dog. Circulation research. 1979;44(5):701–712.

8. Lee AW. A rule-based method for predicting the electrical activation of the heart with cardiac resynchronization therapy from non-invasive clinical data. Medical Image Analysis. 2019;.

9. Nerbonne JM, Kass RS. Molecular Physiology of Cardiac Repolarization. Physiological Reviews. 2005;85(4):1205–1253. doi:10.1152/physrev.00002.2005.

10. Nordsletten DA, Niederer SA, Nash MP, Hunter PJ, Smith NP. Coupling multi-physics models to cardiac mechanics. Progress in Biophysics and Molecular Biology. 2011;104(1-3):77–88. doi:10.1016/J.PBIOMOLBIO.2009.11.001.

11. Guccione JM, McCulloch AD, Waldman LK. Passive material properties of intact ventricular myocardium determined from a cylindrical model. Journal of biomechanical engineering. 1991;113(1):42–55.

12. Nasopoulou A, Shetty A, Lee J, Nordsletten D, Aldo Rinaldi C, Lamata P, et al. Improved identifiability of myocardial material parameters by an energy-based cost function. Biomech Model Mechanobiol. 2017;16:971–988. doi:10.1007/s10237-016-0865-3.

13. Rivlin RS. Large Elastic Deformations of Isotropic Materials. IV. Further Developments of the General Theory. Philosophical Transactions of the Royal Society A: Mathematical, Physical and Engineering Sciences. 1948;241(835):379–397. doi:10.1098/rsta.1948.0024.

14. Di Martino ES, Bellini C, Schwartzman DS. In vivo porcine left atrial wall stress: computational model. Journal of biomechanics. 2011;44(15):2589–2594.

15. Horný L, Žitný R, Chlup H, Macková H. Identification of the material parameters of an aortic wall; 2006. 8. Available from: `https://pdfs.semanticscholar.org/81ae/374f9c206aead1957fb7497b34b419856a6d.pdf`.

16. Tian L, Wang Z, Liu Y, Eickhoff JC, Eliceiri KW, Chesler NC. Validation of an arterial constitutive model accounting for collagen content and crosslinking. Acta Biomaterialia. 2016;31:276–287. doi:10.1016/J.ACTBIO.2015.11.058.

17. Niederer SA, Plank G, Chinchapatnam P, Ginks M, Lamata P, Rhode KS, et al. Length-dependent tension in the failing heart and the efficacy of cardiac resynchronization therapy. Cardiovascular Research. 2011;89(2):336–343. doi:10.1093/cvr/cvq318.

18. Crozier A, Augustin CM, Neic A, Prassl AJ, Holler M, Fastl TE, et al. Image-Based Personalization of Cardiac Anatomy for Coupled Electromechanical Modeling. Annals of biomedical engineering. 2016;44(1):58–70. doi:10.1007/s10439-015-1474-5.

19. Augustin CM, Neic A, Liebmann M, Prassl AJ, Niederer SA, Haase G, et al. Anatomically accurate high resolution modeling of human whole heart electromechanics: A strongly scalable algebraic multigrid solver method for nonlinear deformation. Journal of Computational Physics. 2016;305:622–646. doi:10.1016/j.jcp.2015.10.045.

20. Land S, Gurev V, Arens S, Augustin CM, Baron L, Blake R, et al. Verification of cardiac mechanics software: benchmark problems and solutions for testing active and passive material behaviour. Proceedings of the Royal Society A: Mathematical, Physical and Engineering Sciences. 2015;471(2184):20150641.

21. Westerhof N, Elzinga G, Sipkema P. An artificial arterial system for pumping hearts. Journal of applied physiology. 1971;31(5):776–81. doi:10.1152/jappl.1971.31.5.776.

22. Seed W, Noble M, Walker J, Miller G, Pidgeon J, Redwood D, et al. Relationships between beat-to-beat interval and the strength of contraction in the healthy and diseased human heart. Circulation. 1984;70(5):799–805.

23. Murch SD, Gerche AL, Roberts TJ, Prior DL, MacIsaac AI, Burns AT. Abnormal right ventricular relaxation in pulmonary hypertension. Pulmonary circulation. 2015;5(2):370–375.

24. Pagoulatou S, Stergiopulos N. Evolution of aortic pressure during normal ageing: A model-based study. PloS one. 2017;12(7).

25. Netzer NC, Strohl KP, Högel J, Gatterer H, Schilz R. Right ventricle dimensions and function in response to acute hypoxia in healthy human subjects. Acta physiologica. 2017;219(2):478–485.

26. Strocchi M, Gsell MA, Augustin CM, Razeghi O, Roney CH, Prassl AJ, et al. Simulating ventricular systolic motion in a four-chamber heart model with spatially varying robin boundary conditions to model the effect of the pericardium. Journal of Biomechanics. 2020;101:109645.

27. Fritz T, Wieners C, Seemann G, Steen H, Dössel O. Simulation of the contraction of the ventricles in a human heart model including atria and pericardium. Biomechanics and modeling in mechanobiology. 2014;13(3):627–641.

28. Hyde ER, Behar JM, Crozier A, Claridge S, Jackson T, Sohal M, et al. Improvement of right ventricular hemodynamics with left ventricular endocardial pacing during cardiac resynchronization therapy. Pacing and Clinical Electrophysiology. 2016;39(6):531–541.

29. Land S, Niederer SA. Influence of atrial contraction dynamics on cardiac function. International Journal for Numerical Methods in Biomedical Engineering. 2018;34(3):e2931. doi:10.1002/cnm.2931.

30. Pfaller MR, Hörmann JM, Weigl M, Nagler A, Chabiniok R, Bertoglio C, et al. The importance of the pericardium for cardiac biomechanics: From physiology to computational modeling. Biomechanics and modeling in mechanobiology. 2019;18(2):503–529.
