# Supplementary material for: Linking statistical shape models and simulated function in the healthy adult human heart
Source: PLoS Comput Biol. 2021 Apr 15;17(4):e1008851. doi: 10.1371/journal.pcbi.1008851 (PMC8049237; doi:10.1371/journal.pcbi.1008851)
Supplement: S5 Text — (PDF) [file pcbi.1008851.s005.pdf]

S5 Geometrical quality control

While other mesh quality metrics have been proposed, the SJ metric is both the most common and relevant in the analysis of unstructured meshes [1]. Let $J_k = [e_{1k}, e_{2k}, e_{3k}]$ be the Jacobian matrix evaluated in the vertex k where $e_{ik},\ i = 1, 2, 3$ are the edge vectors of this vertex. The SJ metric is defined for each element e as

$$SJ_e = \min_k \frac{\det J_k}{\|e_{1k}\| \cdot \|e_{2k}\| \cdot \|e_{3k}\|}. \tag{1}$$

This quantity has the range of $[-1, 1]$ where the value of 1 represents a regular tetrahedron, whereas negative values represent a concave (and therefore an inverted and non-valid) element. In Tables A and B these values together with statistics on the elements, nodes and edges are summarised.

References

1. Gao X, Huang J, Xu K, Pan Z, Deng Z, Chen G. Evaluating Hex-mesh Quality Metrics via Correlation Analysis. Computer Graphics Forum. 2017;36(5):105–116. doi:10.1111/cgf.13249.

Mesh #	# Elements ($\times 10^6$)	# Nodes ($\times 10^6$)	# Edges ($\times 10^6$)	EL (mm)	Min. EL (mm)	Max. EL (mm)	SJ value	Min. SJ	Max. SJ
01	2.48	0.49	3.14	1.06 ± 0.15	0.19	1.75	0.73 ± 0.11	0.08	0.99
02	2.77	0.54	3.48	1.06 ± 0.15	0.24	1.72	0.74 ± 0.11	0.09	1
03	2.31	0.46	2.93	1.06 ± 0.15	0.31	1.74	0.73 ± 0.11	0.08	0.99
04	2.7	0.53	3.4	1.06 ± 0.15	0.3	2.49	0.74 ± 0.11	0.07	0.99
05	1.52	0.31	1.95	1.06 ± 0.16	0.38	1.7	0.73 ± 0.11	0.13	0.99
06	1.77	0.36	2.26	1.06 ± 0.16	0.07	2.33	0.73 ± 0.11	0.02	0.99
07	1.73	0.36	2.23	1.06 ± 0.16	0.09	1.74	0.72 ± 0.11	0.04	1
08	2.21	0.45	2.83	1.06 ± 0.16	0.15	1.71	0.73 ± 0.11	0.08	0.99
09	2.05	0.42	2.63	1.06 ± 0.16	0.32	1.74	0.72 ± 0.11	0.3	0.98
10	2.61	0.52	3.31	1.06 ± 0.15	0.23	1.77	0.73 ± 0.11	0.08	0.99
11	2.19	0.44	2.8	1.06 ± 0.15	0.23	1.76	0.73 ± 0.11	0.05	1
12	1.65	0.34	2.12	1.06 ± 0.15	0.26	1.78	0.73 ± 0.11	0.07	0.99
13	2.33	0.47	2.97	1.06 ± 0.15	0.09	1.73	0.73 ± 0.11	0.08	1
14	2.56	0.52	3.26	1.06 ± 0.16	0.14	1.73	0.73 ± 0.11	0.05	0.99
15	1.74	0.36	2.23	1.06 ± 0.16	0.28	1.77	0.72 ± 0.11	0.08	0.99
16	2.03	0.41	2.59	1.06 ± 0.15	0.06	1.75	0.73 ± 0.11	0.03	1
17	1.93	0.39	2.47	1.06 ± 0.15	0.32	1.7	0.73 ± 0.11	0.11	1
18	1.47	0.3	1.89	1.06 ± 0.16	0.3	1.73	0.73 ± 0.11	0.07	1
19	1.93	0.39	2.46	1.06 ± 0.15	0.37	1.76	0.73 ± 0.11	0.1	1
20	1.9	0.38	2.42	1.06 ± 0.15	0.03	1.72	0.73 ± 0.11	0.1	0.99
**Total**	2.09 ± 0.39	0.42 ± 0.07	2.67 ± 0.49	$1.06 \pm 0.15^*$	0.22 ± 0.11	1.8 ± 0.21	$0.73 \pm 0.00^*$	0.08 ± 0.06	0.99 ± 0.00

Table A. The different metrics explained in the text, detailed for each one of the image-based mesh. EL stands for edge length, SJ for scaled Jacobian metric. The number of elements, nodes and edges are in millions, and the edge lengths in mm. In the case of the mean edge length and mean SJ, the total (marked with an *) is the weighted mean ± the weighted SD, where the weights are the number of edges and the number of elements, respectively.

Case	# Elements ($\times 10^6$)	# Nodes ($\times 10^6$)	# Edges($\times 10^6$)	EL (mm)	Min. EL (mm)	Max. EL (mm)	SJ value	Min. SJ	Max. SJ
Average	1.77	0.38	2.7	1.11 ± 0.23	0.08	2.6	0.69 ± 0.13	0.04	1
Mode 1 + 3 SD	1.97	0.41	2.94	1.13 ± 0.24	0.09	3.8	0.69 ± 0.12	0.03	0.99
Mode 1 - 3 SD	2.04	0.42	2.63	1.16 ± 0.23	0.07	3.54	0.69 ± 0.12	0.05	0.99
Mode 2 + 3 SD	2.48	0.5	3.25	1.17 ± 0.21	0.08	3.61	0.7 ± 0.12	0.04	1
Mode 2 - 3 SD	1.63	0.36	2.29	1.03 ± 0.26	0.07	2.7	0.68 ± 0.13	0.06	0.99
Mode 3 + 3 SD	2.14	0.44	2.74	1.16 ± 0.22	0.09	2.93	0.7 ± 0.12	0.05	0.99
Mode 3 - 3 SD	1.65	0.36	2.18	1.07 ± 0.25	0.07	2.68	0.66 ± 0.14	0.04	0.99
Mode 4 + 3 SD	1.77	0.38	2.32	1.12 ± 0.24	0.08	2.96	0.67 ± 0.14	0.03	0.99
Mode 4 - 3 SD	1.85	0.39	2.41	1.11 ± 0.23	0.08	2.63	0.68 ± 0.13	0.08	0.99
Mode 5 + 3 SD	1.8	0.38	2.35	1.11 ± 0.23	0.09	3.09	0.68 ± 0.13	0.03	0.99
Mode 5 - 3 SD	1.83	0.39	2.39	1.11 ± 0.24	0.07	2.76	0.68 ± 0.13	0.06	0.99
Mode 6 + 3 SD	1.86	0.4	2.43	1.11 ± 0.23	0.07	2.46	0.68 ± 0.13	0.09	0.99
Mode 6 - 3 SD	1.71	0.37	2.24	1.12 ± 0.25	0.08	3.44	0.67 ± 0.13	0.02	0.99
Mode 7 + 3 SD	1.69	0.37	2.22	1.1 ± 0.24	0.08	2.55	0.67 ± 0.13	0.06	0.99
Mode 7 - 3 SD	1.89	0.4	2.45	1.12 ± 0.24	0.07	3.14	0.68 ± 0.13	0.06	0.99
Mode 8 + 3 SD	1.81	0.39	2.36	1.1 ± 0.24	0.08	2.46	0.68 ± 0.13	0.08	0.99
Mode 8 - 3 SD	1.76	0.38	2.3	1.12 ± 0.24	0.08	3.04	0.68 ± 0.13	0.05	0.99
Mode 9 + 3 SD	1.92	0.41	2.49	1.12 ± 0.23	0.08	2.8	0.68 ± 0.13	0.04	1
Mode 9 - 3 SD	1.62	0.35	2.14	1.11 ± 0.24	0.08	2.69	0.67 ± 0.13	0.08	0.99
**Total**	1.85 ± 0.2	0.39 ± 0.03	2.46 ± 0.28	$1.12 \pm 0.24^*$	0.08 ± 0.01	2.94 ± 0.4	$0.68 \pm 0.01^*$	0.05 ± 0.02	0.99 ± 0.0

Table B. The different metrics explained in the text, detailed for each mesh of the extreme3 cohort. EL stands for edge length, SJ for scaled Jacobian metric. The number of elements, nodes and edges are in millions, and the edge lengths in mm. In the case of the mean edge length and mean SJ, the total (marked with an *) is the weighted mean ± the weighted SD, where the weights are the number of edges and the number of elements, respectively. The table corresponds to the volumetric meshes derived from the topologically consistent surfaces (the surfaces all had the same number of elements).
