# Supplementary material for: Linking statistical shape models and simulated function in the healthy adult human heart
Source: PLoS Comput Biol. 2021 Apr 15;17(4):e1008851. doi: 10.1371/journal.pcbi.1008851 (PMC8049237; doi:10.1371/journal.pcbi.1008851)
Supplement: S6 Text — (PDF) [file pcbi.1008851.s006.pdf]

S6 Interpreting mode 9

In the LV, mode 9 is specifically related to the localised basal septal hypertrophy, which is a known marker of increased afterload and localized deformation impairment [1]. This morphological trait is mainly defined from ratios between several wall thickness measurements. The recognition of the localised hypertrophy allows for the identification of the hypertensive patients in need of a closer clinical follow-up.

Although the meaning of the modes is not straight forward to define, we can interpret them analysing the displacement of the meshes of the extreme3 cohort with respect to the average mesh. In Fig A we show the displacements of mode 9 scaled, as well as a contour of the LV.

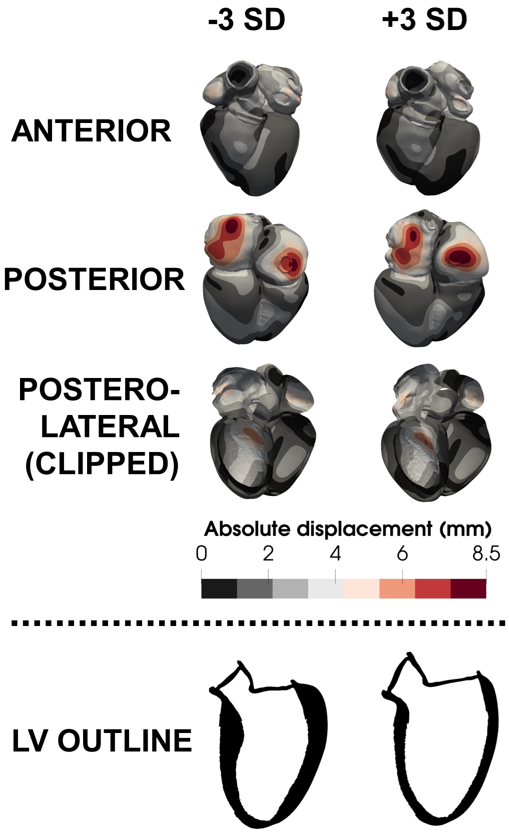

Figure A. Visualisation of mode 9. Meshes correspond to the extreme3 cohort colour-coded with their distance with respect to the average mesh. We generated an extra view of the LV septum with a different scale to highlight the change in septal anatomy associated with modifications to this mode.

References

1. Loncaric F, Nunno L, Mimbrero M, Marciniak M, Fernandes JF, Tirapu L, et al. Basal Ventricular Septal Hypertrophy in Systemic Hypertension. The American Journal of Cardiology. 2020;.
